# High-Frequency Pulsatile Parameterization Study for the Titania Ceramic Membrane Fouling Mitigation in Oily Wastewater Systems Using the Box–Behnken Response Surface Methodology

**DOI:** 10.3390/membranes12121198

**Published:** 2022-11-28

**Authors:** Mohamed Echakouri, Amr Henni, Amgad Salama

**Affiliations:** Process Systems Engineering, Produced Water Treatment Laboratory, Faculty of Engineering and Applied Science, University of Regina, Regina, SK S4S 0A2, Canada

**Keywords:** oily wastewater, titania ceramic membrane, fouling control, pulsatile flow, parameterization, response surface methodology, statistical modeling, regression model

## Abstract

In this comprehensive study, a seven-channel ultrafiltration (UF) titania membrane was used to investigate the impact of the pulsatile cleaning process on the crossflow filtration system. Seventeen experimental runs were performed for different operating conditions with a transmembrane pressure (TMP) varying from 0.5 to 1.5 bar, a crossflow velocity (CFV) ranging from 0.5 to 1 m/s, and pulsatile parameters within an interval varying from 60 to 120 s with a duration of 0.8 s, and collecting membrane permeate flux and volume data. The optimized operating conditions revealed that a TMP of 1.5 bar, a CFV of 0.71 m/s, and a pulsatile cycle of 85 s were the best operating conditions to reach the highest steady permeability flux and volume of 302 LMH and 8.11 L, respectively. The UF ceramic membrane under the optimized inputs allowed for an oil-rejection ability of 99%. The Box–Behnken design (BBD) model was used to analyze the effect of crossflow operating conditions on the permeate flux and volume. The analysis of variance (ANOVA) indicated that the quadratic regression models were highly significant. At a 95% confidence interval, the optimum TMP significantly enhanced the flux and permeate volume simultaneously. The results also demonstrated a positive interaction between the TMP and the pulsatile process, enhancing the permeate flux with a slight impact on the permeate volume. At the same time, the interaction between the CFV and pulsatile flow improved the permeability and increased the permeate volume.

## 1. Introduction

Produced water (PW) is a significant byproduct of the oil and gas industry. PW is a mixture of dispersed oil and other hydrocarbons, dissolved inorganic and organic compounds, corrosion and treatment chemicals, heavy metals, solids, and residual production-line additives [1,2]. Produced water typically encompasses toxic pollutants, posing a tremendous environmental threat and precluding additional production activities. Taking tangible steps to treat or reuse produced water for sustainability and environmental preservation is becoming indispensable. However, PW composition and properties vary greatly based on various parameters, including the geographic field location, reservoir extraction age, reservoir geological formation, exploitation method, type of production facilities, and reservoir composition [3]. Membrane filtration has become a promising technique to meet the new environmental regulations and laws compared to earlier conventional treatment methods, such as adsorption, chemical oxidation, chemical precipitation, electrodialysis, evaporation, air flotation, gravity separation, and hydrocyclones [4]. However, membrane fouling is a significant drawback to membrane filtration due to the accumulation of oil droplets on the membrane surface and within the membrane pores. Fouling development causes concentration polarization and enhances the formation of the cake layer, which reduces the membrane performance and accelerates its deterioration [5]. Operating the membrane filtration system below the critical flux will significantly mitigate membrane fouling. Critical permeation flux is the flux at which the oil droplets’ convection towards the membrane surface and their back-diffusion forces to the bulk are equal. Nevertheless, operating the membrane under the subcritical flux generates a low permeate flow [6,7].

Numerous methods have been used to control membrane fouling in crossflow filtration systems. The optimization of the operating conditions (i.e., crossflow velocity and transmembrane pressure) [8], feed turbulence promoters (i.e., reducing the concentration polarization) [9], high shear crossflow, and membrane module-surface hydrodynamic modification are examples of the conventional methods used to combat fouling development. Membrane cleaning methods have also been extensively used for fouling control; cleaning techniques can be categorized into chemical and physical processes. Chemical cleaning uses agents (e.g., alkalines and acids) to regenerate the membrane surface and clean the irreversible membrane fouling [10]. Physical cleaning uses mechanical and hydrodynamic forces to help displace and transport the oil droplets by the crossflow mainstream [11]. Mechanical cleaning techniques include ultrasonic and vibration [12]. Hydrodynamic cleaning consists of backflush pulsing using forward washing, pneumatic (air flushing, airlifting, and air bubbling), and feed pressurizing/depressurizing techniques [13].

The proposed Back-Pulsatile (BP) process (or Back-Pulsing) is a promising in situ physical antifouling technique for ceramic membrane fouling mitigation during crossflow filtration. Pulses are cyclically generated when the transmembrane pressure reaches a critical level under a constant permeate-flux filtration or at a specific time interval to reverse the filtration, loosen, and displace the oil droplets present at the membrane surface and in its pores [14]. The use of pulses in crossflow involves switching the TMP typically for one second or less at a high frequency of approximately 0.1 to 2 Hz [15]. Many research groups have extensively studied back-pulsing efficiency in crossflow filtration to control and mitigate membrane fouling while maintaining high permeation flux over the filtration time. Salalahi et al. [16] evaluated the effect of feed parameters (e.g., oil content and oil composition) and membrane flux on the efficiency of back-pulsing on ceramic-membrane filtration performance. The results showed that applying the back-pulsing significantly minimizes the fouling rate and improves the membrane performance. Gao et al. [17] examined the back-pulsing parameters (amplitude, duration, and frequency) and their interactions with membrane performance. The findings demonstrated that amplitude was the most critical variable for fouling clearance and the overall net-specific flux. However, the frequency was found to be the most important variable for the membrane’s net production. Borujeni et al. [18] evaluated the performance of hollow-fiber ultrafiltration membranes at various back-pulse frequency, amplitude, and duration settings. Their results reveal that the back-pulsing operation throughout a filtration process can yield a constant flux with a substantially higher permeate-solution recovery. Ramirez et al. [19] tested the rapid back-pulsing effectiveness in improving the dilute oil-in-water clay-membrane crossflow microfiltration. The experimental results showed that the rapid back-pulsing presented high efficacy in enhancing the permeate flux approximately 25 times under an optimum duration and frequency. However, they recommended coupling the back-pulsing with other techniques (e.g., direct firing of the ceramic membrane) to maintain a higher membrane performance. Mores et al. [20] also investigated the effect of rapid back-pulsing in crossflow microfiltration on membrane performance. Direct visual observation has been accomplished to determine the development of the membrane surface fouling. The results revealed that the membrane was cleaned efficiently under a longer back-pulsing duration and higher back-pulse pressure.

The pulsatile method for treating the membrane fouling in the crossflow mode has been studied under the influence of the pulsatile parameters (amplitude, duration, and frequency). However, there are significant scientific gaps in the design studies of combining crossflow and pulsatile parameters to mitigate membrane fouling as the pulsatile process is implemented in crossflow filtration. A solid experimental design methodology should be implemented to shed light on the impact of crossflow, transmembrane pressure, back-pulse frequency, amplitude, and duration on the membrane performance.

In this study, we have assessed the crossflow filtration with pulsatile cleaning for the treatment of synthetically produced water prepared with oil from the Bakken reservoir (Southern Saskatchewan, Canada) using a ceramic membrane with an active layer of titania and a supporting layer of zirconia. Experiments were performed to measure the impact of pulsatile process and crossflow parameters (crossflow, transmembrane pressure, interval/frequency, and duration) on the membrane’s overall net flux enhancement and membrane fouling control for a feed oil content of 200 ppm, which is the concentration of the Bakken reservoir-produced water after initial treatment.

A Box–Behnken response surface approach [21,22,23] was used to identify factors affecting the membrane fouling and to optimize the overall permeate flux. Three independent factors were carefully studied: the crossflow velocity (0.5 to 1.5 m/s) [24]; transmembrane pressure (0.5 to 1.5 bar) [25]; and pulsatile time interval (60 to 120 s) at a constant pulse duration (0.8 s). The overall permeate volume was collected, and its oil content and turbidity were analyzed for membrane rejection performance. The independent variable interactions and their impact on the membrane fouling were investigated to quantify their effects on the permeate flux and overall permeate volume. Finally, the pulsatile reversal flow mechanism was discussed to improve the understanding of this physical cleaning technique. The pulsatile flow efficiency was also examined to investigate the membrane performance with versus without the pulsatile process.

## 2. Experimental Methodology

In this study, a LabBrain filtration unit manufactured by LiqTech International (Figure S1 (Supplementary Materials)) was used to perform all the experiments in the crossflow mode. The experiments were operated in batch mode, where the feed/concentrate is continuously recirculated through the ceramic membrane for 2 h. The permeate was collected, and reverse osmosis water was added to the feed to maintain the feed concentration constant. The filtration unit setup automatically logged all operational conditions such as transmembrane pressure (TMP), crossflow velocity (CFV), retentate temperature, the valve opening percentages, permeate flow rate, retentate flow rate, and feed flow rate every 3 s. For each experiment, a synthetic feed of 24 L of produced water was prepared and immediately used. The feed and permeate were characterized by measuring their oil content. Additional feed characterization was performed to measure the feed mean droplet size, zeta potential, chemical oxygen demand (COD), turbidity (TNU), pH, density, and viscosity. The ceramic membrane morphology, pore size, molecular weight cut-off (MWCO), retention capability, permeability, ceramic membrane geometry/dimensions, and thermal/chemical resistance were all reported. After each experiment, the filtration unit was meticulously washed/drained by circulating RO water to remove any impurities/ pollutants. At the end of the experiment, the ceramic membrane was cleaned using RO water (at 50 °C), Sodium hydroxide (at 50 °C), and Phosphoric acid (at 85 °C), respectively, reaching a flux recovery of 99%. The purities of the chemicals used are reported in Table 1.

### 2.1. Materials

Bakken light oil from Southern Saskatchewan (Canada) was used in the experiments. It has a density of 0.8872 g/cc and a viscosity of 5.23 cP. Reverse osmosis (RO) was filtered by an ultraviolet (UV) water purification system (EMD Millipore, Burlington, MA, USA, 2012) to prepare ultrapure deionized water (DI, <5 ppb TOC and <0.1 colony-forming units of a microorganism/mL). Sodium dodecyl sulfate (SDS, 99 wt.% pure) was purchased from Sigma-Aldrich (St. Louis, MO, USA) and used for stability in feed preparation. The 7-channel ceramic membrane was purchased from Tami industries and cut into 25 × 305 mm pieces. Additional chemicals (Table 1) were purchased and used as received for the ceramic membrane cleaning or oil/solvent extraction.

### 2.2. Feed Synthesis and Characterization

The synthetic-produced water feed of 200 ppm (Figures S2 and S3) was prepared by adding 4.5 mL of Bakken oil in 2 L batch of reverse osmosis water and 0.3 mM Sodium dodecyl sulfate (SDS) for feed stability. The light oil properties were measured using an Anton Paar 5000 DSA digital densitometer and a Brookfield viscometer DV-II + Pro at 22.5 °C, giving a density of 0.8872 g/cc (±5 × 10^−5^ g/cc accuracy) and a viscosity of 5.23 cp (±1.0% accuracy). The 2 L oily water emulsion was mixed for 2 min at 18,000 rpm using a commercial blender to homogenize its composition and ensure feed stability. This process was repeated to prepare a total volume of 24 L (12 batches) for each experiment. Additional feed properties (Table 2) were measured using the instruments listed in Table 3.

### 2.3. Ceramic Membrane Characterization

**Figure 1:**
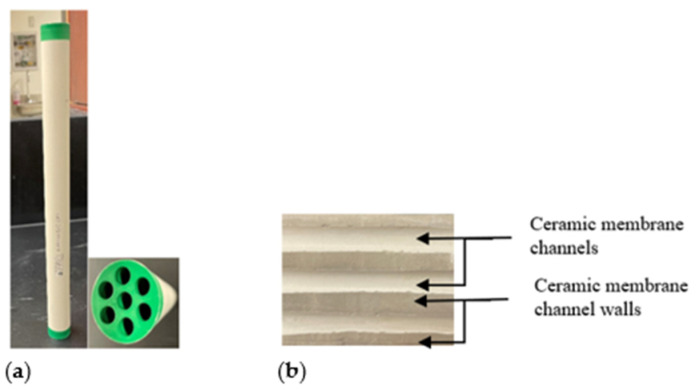
Ceramic membrane picture used in the experiment: (**a**) the entire illustration and cross-sectional area, (**b**) ceramic membrane internal channels and walls.

This study used an ultrafiltration ceramic membrane (zirconia/titania) with seven channels (Figure 1) for the produced water treatment using the LabBrain filtration unit. Table 4 provides the essential membrane properties and specifications. The ceramic membrane active filtration and cross-sectional area were found to be 0.04186 ± 0.006 and 0.001172 ± 0.006 m^2^, respectively. The synthetic-produced water emulsion meant that the oil droplet size was measured with a Zetasizer to be around 5.2 ± 0.1 μm. In addition, the oil and water droplet adsorptions on the ceramic membrane surface were performed to measure the surface wettability responses. Figures S4 and S5 illustrate the membrane surface oleophobicity and super-hydrophilicity characteristics with contact angles of 135 and 35°, respectively.

### 2.4. Crossflow Filtration System Description

The LabBrain membrane crossflow filtration system (Figure S6) manufactured by LiqTech International (Hobro, Denmark) was used to run all the experiments under batch mode conditions. Figure S6 shows a drawing of the LabBrain Proportional and Integral (P&I) control-loop diagram system. The LabBrain unit was outfitted with a membrane housing with a ceramic element measuring 25 × 305 mm ± 1 mm. The filtration unit valves were controlled by a PLC (Siemens 6ES7 214-1AE30-OXBO, Siemens, Munich, Germany) and fed by a MotoMaster air compressor of 2.5 US gallons capacity operating at a pressure of 6 bar. The feed circulation was performed by a pump (Grundfos CRN 3–6, Grundfos, Bjerringbro, Denmark) with a 5 m^3^/h capacity at 2.5 bar. When the Data log was enabled, all parameters relating to the valves’ actual openings, pressure and flow transmitters, pump speed, transmembrane pressure, flux, and production were recorded in the unit’s internal memory every 3 s.

Before the start of the experiment, the ceramic membrane was progressively immersed in deionized water and then steeped for twelve hours to remove the trapped air in the membrane and to enhance the overall permeability of water flow. The membrane element was then mounted and sealed inside the housing cell. The feed emulsion was synthesized and poured into the oily wastewater tank to run the experiment. The pulsatile permeate was then collected and quantified, and the retentate and permeate were fully returned to the feed tank. Feed concentration was stably maintained by constantly adding reverse osmosis water to the container in the same sampled permeate volume during the two-hour experiment.

The LabBrain filtration unit worked at settings of 1 m/s crossflow (CFV) and 1.5 bar trans-membrane (TMP). The speed of the pump and concentrate valve were progressively adjusted. Once the pressure in the unit reached a steady level, data were recorded. At the end of the experiment, a 4 mL permeate sample was characterized by measuring its oil content and turbidity.

## 3. Box–Behnken Design

The Box–Behnken design (BBD) [26,27,28] was used to study the impact of the pulsatile process in crossflow filtration mode on the membrane fouling control and mitigation. The combination of the crossflow operating conditions and pulsatile parameters was selected to understand their influence on the membrane performance. The independent process variables were the TMP, CFV, and pulsatile parameters (BP cycle). The range of the independent factors was determined based on the configuration of the experimental setup and initial single-variable experiments [6]. The pulsatile duration was constant at 0.8 s, the BP cycle varied from 60–120 s, the TMP range of 0.5 to 1.5 bar, and CFV from 0.5 to 1.0 m/s. Table 5 represents the independent factors and their coded levels −1, 0, and 1 at high, middle, and low, respectively. Permeate flux (LMH) and permeate volume (L) were selected as responses to the B-B design (Table 6). Seventeen experiments were randomly generated with 5 central replications points and 12 design points (Table 7). In Equation (1), *N* is the total number of experiments, *k* denotes the number of investigated factors, and *C*_0_ is the number of central replication points:

(1) $$N=2k\;\left(k-1\right)+C_{0}$$

The Design Expert program (v.12) was used to analyze the responses using a quadratic polynomial regression model (Equation (2)) to fit and optimize the experimental data:

(2) $$Y_{k\;}=b_{0}+\overset{n}{\underset{i=1}{\sum }}b_{i}\;x_{i\;}+\overset{n}{\underset{i=1}{\sum }}b_{ii}\;x_{i}^{2}+\overset{n}{\underset{i<j}{\sum }}b_{ij}\;x_{i}\;x_{j\;}+S_{e\;}$$

where *Y_k_* is the predicted response, namely *Y*_1_ for membrane permeate flux and *Y*_2_ for membrane permeate volume. *x_i_* denotes the coded variables, b_0_ is a model constant, and *S_e_* is the statistical error. In the equation, *b_i_*, *b_ii_*, and *b_ij_* are the linear, quadratic, and interactional regression coefficients, respectively. The model coefficients were estimated by multiple regression analysis. The polynomial model fitness was conducted using the lack of fit and the coefficient of determination (R^2^).

The experimentally examined responses were *J_ni_* (final steady permeate flux, in LMH) based on Equation (3):

(3) $$J_{i}=J_{0}\;,\;\;at\;t=0\;\min\;J_{i}=J_{ni}\;,\;at\;t=120\;\min$$

where *J_i_* is the flux at any time; and *J*_0_ is the initial flux of the membrane filtration (Table S1).

*Y_ni_* is the experiment’s final net permeate volume measured in liters (L) calculated using Equation (4).

(4) $$Y_{ni}=Total\;amount\;of\;permeate-Total\;pulsatile\;water\;consumption$$

The membrane rejection was calculated using Equation (5):

(5) $$Rejection\;\left(\%\right)=\left(1-\frac{C_{p}}{C_{f}}\;\right)\times 100\;$$

where *C_p_* and *C_f_* are the permeate and the feed oil content, respectively, in ppm; the membrane performance and fouling control were measured in each experimental run based on the permeate flux decline, overall permeate volume (Table 7), rejection capacity, and permeate turbidity (Table 8).

## 4. Results and Discussion

Figure S7 depicts the normal filtration and pulsatile flux–decline patterns. The decline in the permeate flux can be categorized into three sections. In the first section, the flux drops from its highest value at the early filtration time before reaching a pseudo-stabilized stage. Normal filtration flux decline is more significant than pulsatile flux initial decay. Consequently, the membrane surface was rapidly covered by oil droplets if the pulsatile cycle was initiated. In normal filtration mode, the crossflow field could not evacuate and transport oil droplets driven by the permeation drag field [29]. While under the pulsatile process, the decline was not very pronounced at the early stages due to the additional periodic pulsatile-cleaning impact.

The second section showed a pseudo hydrodynamic balance between crossflow cleaning and fouling development. In normal filtration mode, at 60% of initial flux, the permeate flux started to flatten until reaching a low steady-state permeate flux of 35 LMH at the final stage. Similarly, the flux decline was less manifest in the pulsatile process than in the normal filtration. The best performance of the pulsatile process was found to be for a permeate flux of 298 LMH under a periodic pulsing of a 60-s cycle, a TMP of 1.5 bar, and a CFV of 0.75 m/s. A high pulsatile permeate flux decline of 85 LMH was observed in a pulsatile cycle of 90 s, TMP of 0.5 bar, and CFV of 0.5 m/s.

Table 7 depicts that an increase in the TMP to a level of 1.5 bar enhances the permeate flux and net permeate volume (Runs 1, 4, and Runs 2, 3). In addition, a high transmembrane pressure of 1.5 bar and a pulsatile reversal-flow level of 90 or 60-s lead to a high membrane performance (Runs 1, 3, 8, and 11). The central point runs (Runs 9, 12, 14, 15, and 17) at a pulsatile cycle of 90-s show a better membrane performance than all pulsatile cycles of 120 s configurations.

At the high pulsatile flow sequence of 60-s, the increase in CFV enhanced the membrane performance (Runs 5 and 6). In contrast, the increased CFV did not improve the membrane performance at the low pulsatile frequency of 120 s. As a result, we conclude that a combination of the CFV and pulsatile flow plays a crucial role in mitigating membrane fouling. This cleaning process prevents the oil droplets, dragged by the permeation flux, from aggregating at the membrane surface and displacing them by the crossflow field, which maintains the cleanliness of the membrane surface and combats the membrane fouling.

In conclusion, the membrane steady-state permeate flux was enhanced, reaching a value of 298 LMH using the pulsatile cycle compared to a normal filtration value of 35 LMH. This can be explained by the contribution of the pulsatile process as an additional implemented physical cleaning process to combat membrane fouling and enhance membrane performance (Table 7).

Rejection improved from 92% to 99% by applying the pulsatile process. The impact of the pulsatile cycle for each run demonstrated that oil rejection for all configurations was better than in the normal filtration mode. The reduction in permeate turbidity was also enhanced from 4.58 NTU to 0.32 NTU when using this physical cleaning process (Table 8).

### 4.1. Box–Behnken Model

The Box–Behnken design method was used to model the 17 runs for both the experimental and predicted results, as reported in Table 3. Second-order regression polynomial equations were generated to elucidate the liaison between the membrane permeate flux/permeate volume and design-independent factors. The predictive response models were defined in coded and actual factors (Equations (6)–(9)) [30]. A list of coefficients are presented in Table 9 and Table 10. The coefficient correlation value represented the corresponding factor unit change when all other factors were maintained as constant. In contrast, the intercept estimated the overall mean response of all 17 runs [31].

The following are the full response quadratic regression equations in terms of coded factors before reduction:

(6) $$Y_{Flux\;}=249.23+86.59\;A+11.13\;B-5.80\;C-3.87\;AB-20.32\;AC-43.54\;BC-44.31\;A^{2}-15.29\;B^{2}-22.06\;C^{2}\;\;\;\;\;\;$$

(7) $$Y_{Permeate\;}=8.23-0.439\;A-0.180\;B-0.046\;C-0.038\;AB+0.005\;AC-0.078\;BC+0.313\;A^{2}-0.415\;B^{2}-0.258\;C^{2}\;\;\;$$

The obtained coefficients in Equations (6) and (7) were used for response prediction regarding flux and permeate volume, respectively. A response value was estimated for each combination of coded factor levels (1, 0, −1). In contrast, the polynomial factor coefficients identified the amplitude factor impact on the corresponding response. For the actual response quadratic regression Equations (8) and (9), the response value was predicted using the actual level unit values (TMP: 1.5,1, 0.5; CFV: 1, 0.75, 0.5; and Pulsatile cycle: 120, 90, 60), for each factor.

The full response quadratic regression equations in terms of actual factors before reduction are:

(8) $$y_{Flux\;}\left(LMH\right)=-990.24+672.80\;TMP+964.79\;\;CFV+9.93\;BP\;cycle\;-30.96\;TMP\times CFV-1.35\;TMP\times BP\;cycle\;-5.81\;CFV\times BP\;cycle-177.58\;TMP^{2}-244.58\;CFV^{2}\;-0.03\;BP\;cycle^{2}$$

(9) $$y_{Permeate\;}\left(L\right)=4.091-3.183\;TMP+10.470\;CFV+0.057\;BP\;cycle\;-0.300\;TMP\times CFV+0.0003\;TMP\times BP\;cycle\;-0.010\;CFV\times BP\;cycle+1.250\;TMP^{2}-6.640\;CFV^{2}\;-0.0003\;BP\;cycle^{2}$$

**Figure 2:**
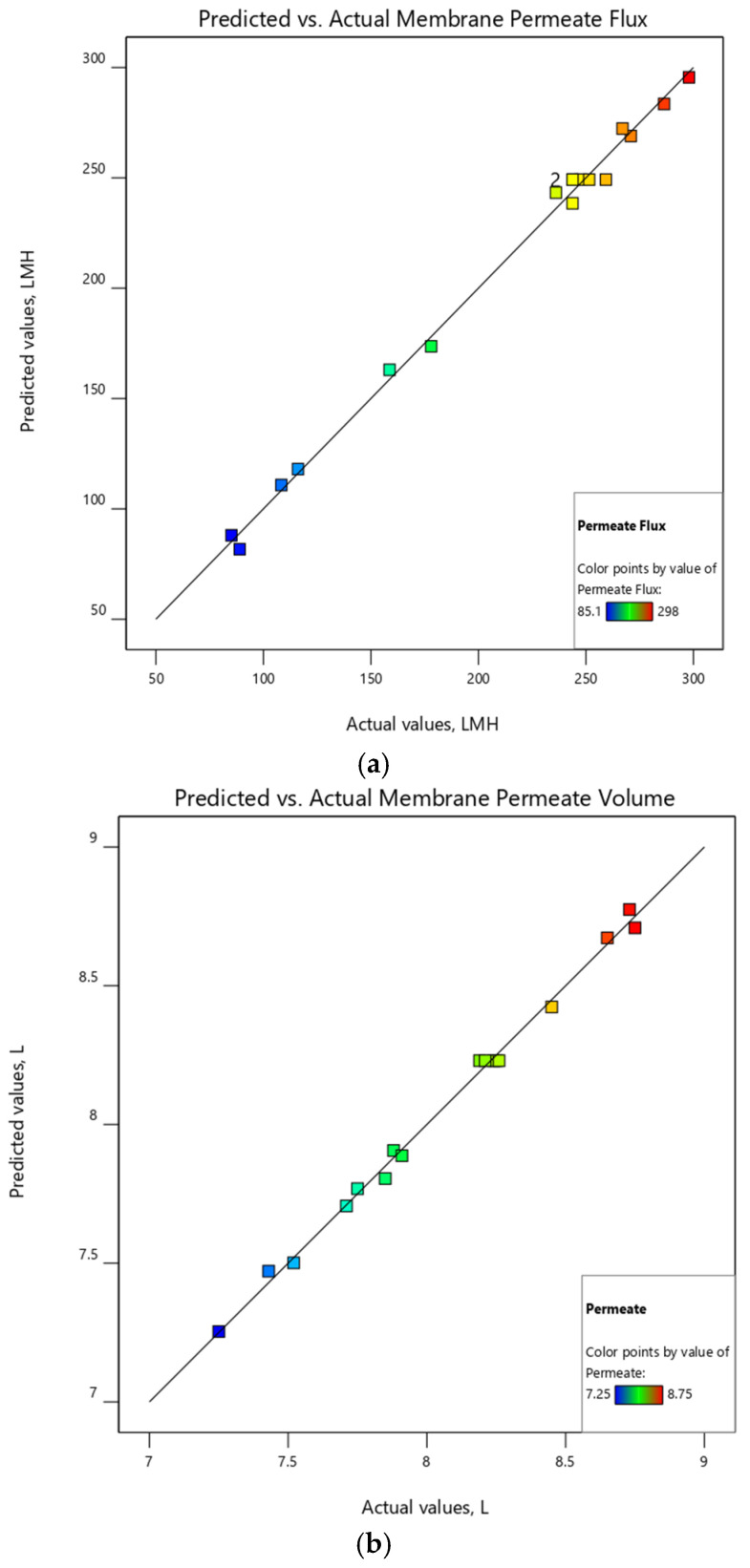
Membrane predicted values versus the actual value. (**a**) Permeate-predictive flux model and (**b**) permeate-predictive volume model for the experiment.

The predictive model graphs for the membrane flux and permeate volume were illustrated in Figure 2a,b, respectively. The positive linear regression between all sets of experimental data for flux and permeate volume and the responses of the second-order regression polynomial equations showed that the prediction models approximated the actual data very well. The fitting statistical results between the actual and predicted values are shown in Table 11. The high values of the coefficients of determination (R^2^) of 0.995 and 0.996 for the flux and permeate, respectively, prove a strong correlation between the experimental and predicted data. The predictive R^2^ of 0.952 and 0.946 show excellent agreement with the adjusted R^2^ of 0.989 and 0.990 for the flux and permeate models, respectively. The adjusted R^2^ of 0.989 and 0.990 shows a high predictive capability of the two regression models for the flux and permeate volume, respectively [32].

### 4.2. Analysis of Variance (ANOVA) for the Quadratic Model

The regression analysis results and the interaction between the actual factors were performed using ANOVA for membrane flux and permeate volume (Table 12 and Table 13). The ANOVA of the two quadratic polynomial models was highly significant in fitting the regression models. The large model F-values of 159.63 and 179.28 indicated that there was only a 0.01% chance (*p*-value < 0.0001) that a higher F-value might occur due to noise for both models dealing with the membrane flux and permeate volume, respectively. The *P*-values of the two models were 0.0001, a very small value compared to the significance level (α = 0.05); this implies that the quadratic regression model equations showed a high goodness level of fit of the experimental response data.

For the membrane permeate-flux goodness of fit model, at a 95% confidence level, the terms with *p*-values less than 0.05 were significant model terms. In this scenario, *A*, *B*, *AC*, *BC*, *A*², *B*², and *C*² were considered. Similarly, the permeate volume model’s significant terms were *A*, *B*, *C*, *BC*, *A*², *B*², and *C*² [33].

The F-value was used to rank the significant regression factors. The significant terms are associated with higher F-values. The following are the rankings of significant regression terms for the two responses models based on F-values:

Membrane permeate flux model: *A* > *A*^2^ > *BC* > *C*^2^ > *AC* > *B* > *B*^2^

Membrane permeate-volume model: *A* > *B*^2^ > *A*^2^ > *C*^2^ > *B* > *BC* > *C*.

After the elimination of the non-significant interaction terms (*p*-value > 0.05), the final simplified-response quadratic regression models at a 95% confidence level were as follows:

The full simplified response quadratic regression equations in terms of coded factors after reduction are:

(10) $$Y_{Flux}^{\prime }=249.23+86.59\;A+11.13\;B-20.32\;AC-43.54\;BC-44.31\;A^{2}-15.29\;B^{2}-22.06\;C^{2}$$

(11) $$Y_{Permeate}^{\prime }=8.23-0.439\;A-0.180\;B-0.046\;C-0.078\;BC+0.313\;A^{2}-0.415\;B^{2}-0.258\;C^{2}$$

The full simplified response quadratic regression equations in terms of actual factors after reduction are:

(12) $$y_{Flux}^{\prime }\;\left(LMH\right)=-990.24+672.80\;TMP+964.79\;\;CFV\;-1.35\;TMP\times BP\;cycle-5.81\;CFV\times BP\;cycle\;-177.58\;TMP^{2}-244.58\;CFV^{2}-0.03\;BP\;cycle^{2}$$

(13) $$y_{Permeate}^{\prime }\left(L\right)=4.091-3.183\;TMP+10.470\;CFV+0.057\;BP\;cycle\;-0.010\;CFV\times BP\;cycle+1.250\;TMP^{2}-6.640\;CFV^{2}\;-0.0003\;BP\;cycle^{2}$$

For all the experimental runs, the simplified quadratic regressions’ Equations (10) and (11) in terms of coded factors, and Equations (12) and (13) in terms of actual factors were used to predict the permeate flux and net permeate volume, respectively.

### 4.3. Lack of Fit Value

As reported at the bottom of Table 12 and Table 13, the results of lack of fit were not significant for both regression models relative to the pure error. The F-value for the flux model was 1.88; this implied the model’s high capability to predict the experimental data variation. Similarly, the lack of fit F-value for the permeate volume model was 4.15, indicating the model’s ability to estimate the considered empirical data variance [34]. In addition, Table 12 and Table 13 illustrated that the relative *p*-values for both responses, flux and permeate volume, were 0.2747 and 0.1015, respectively, which were higher than 0.05. This meant that the models correlated with the experimental data very well, and there was a non-significant lack of fit [35].

### 4.4. Residual Analysis

**Figure 3:**
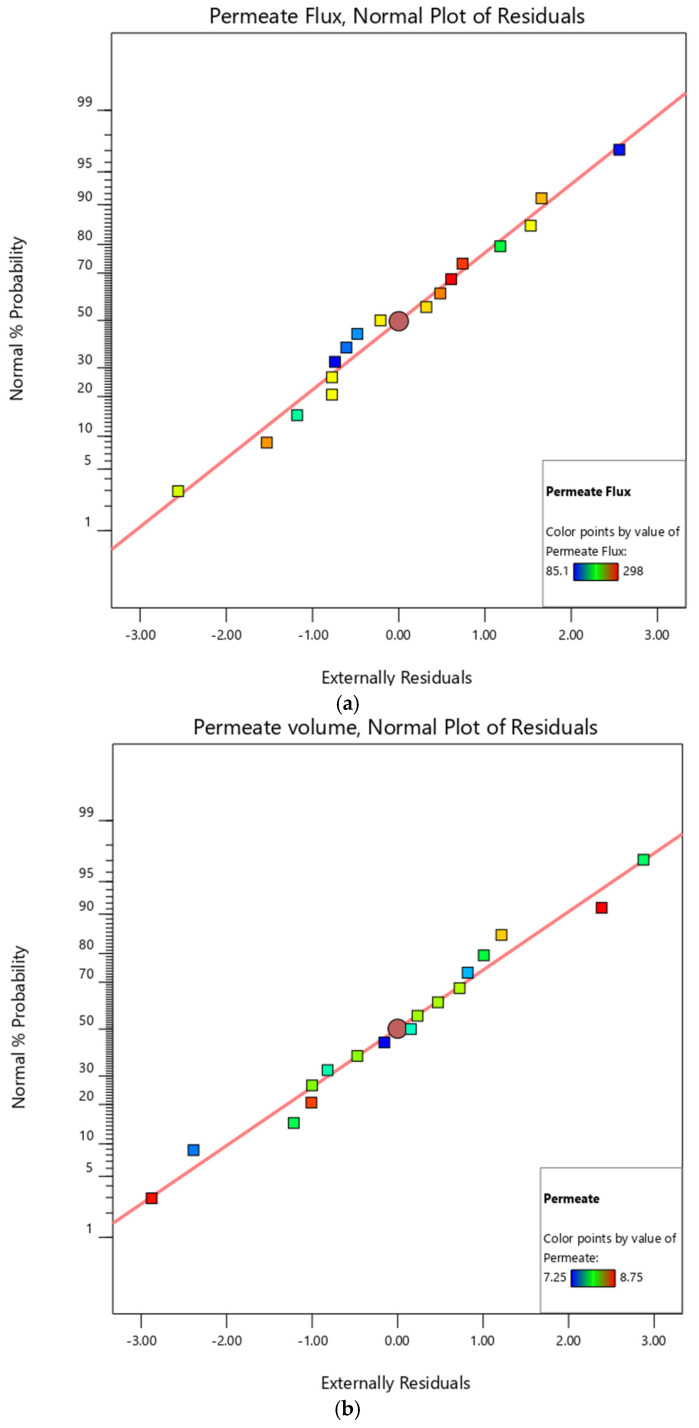
Diagnostic normal probability plots of residuals for (**a**) permeate flux, and (**b**) membrane permeate volume.

The average probability plot residuals can also assess the model quality of fit. The plots in Figure 3 provide valuable statistical evidence. The deviation between the observed and predicted values is referred to as the residual. The normal plot of residuals is an excellent indicator of the correctness of the overall model represented by Equations (6)–(9), and defined by the Box–Behnken experimental design. The residuals in the normal probability plot were linear, indicating that the residuals satisfied the normal distribution assumption. As a result, all predicted and experimental results were in excellent agreement, validating the significance of the quadratic models presented in Equations (10)–(13) [32,33,35,36,37].

### 4.5. Factors Interaction Effect and Membrane Fouling

**Figure 4:**
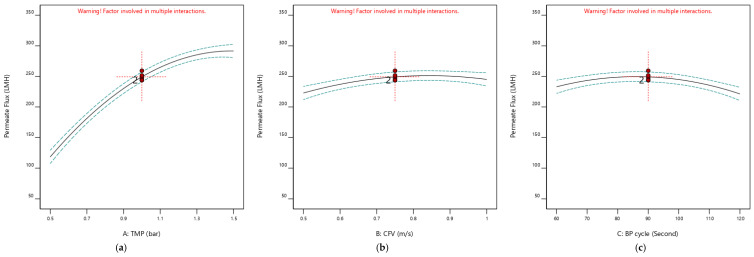
All independent factors (TMP, CFV, and Pulsatile cycle) perturbation for permeate flux considering one factor at time impact. (All design points are at 95% CI bands). (**a**) B: CFV = 0.75 m/s and C: BP cycle = 90 s. (**b**) A: TMP = 1 bar and C: BP cycle = 90 s. (**c**) A: TMP = 1 bar and B: CFV = 0.75 m/s.

**Figure 5:**
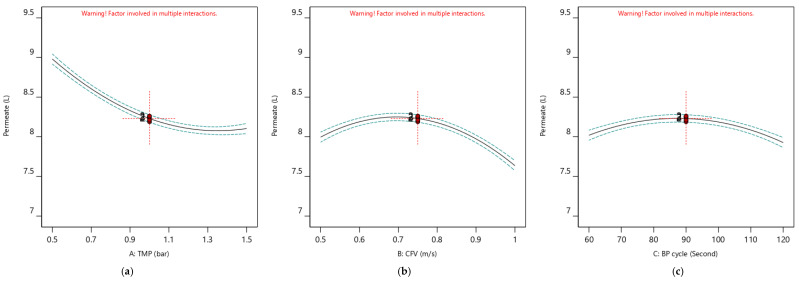
All independent factors (TMP, CFV, and Pulsatile cycle) perturbation for membrane permeate volume considering one factor at time impact. (All design points are at 95% CI bands). (**a**) B: CFV = 0.75 m/s and C: BP cycle = 90 s. (**b**) A: TMP = 1 bar and C: BP cycle = 90 s. (**c**) B: CFV = 0.75 m/s and A: TMP = 1 bar.

The impact of the operating parameters (TMP, CFV, and pulsatile cycle) on the membrane permeate flux and permeate volume are presented in Figure 4 and Figure 5, respectively. The factors’ perturbation effects and their deviation from the reference configuration point (TMP: 1 bar, CFV: 0.75 m/s, and Pulsatile cycle: 90 s) are illustrated. Figure 4 demonstrates the responses’ variation as a one-factor level with changes from low to high: −1, 0, +1, while the other factors were maintained constant at their specific levels. Each factor used in this experiment had a distinct impact on the membrane permeate flux and the overall permeate volume. When the CFV and Pulsatile cycle were held at their optimum level (coded value = 0), the TMP dominated the response values. The increase in the membrane permeate flux is proportionally associated with the increase of the TMP factor from 0.5 bar (coded value = −1) to 1.5 bar (coded value = +1), as shown in Figure 4a; in comparison, the membrane permeate volume stabilized at its optimum value with the increase of the TMP from 0.5 bar (coded value = −1) to 1.5 bar (coded value = +1) (Figure 5a). One should consider that a part of the permeate volume was used continuously for cleaning to maintain the membrane surface, which ensures a higher permeate flux.

The TMP and Pulsatile cycle were maintained at their middle levels, of 1 bar, and 90 s, respectively. The membrane permeate flux and permeate volume values improved with the increase of CFV from 0.50 cm/s (coded value = −1) to 0.75 cm/s (coded value = 0) (Figure 4b and Figure 5b). Similarly, the pulsatile level change from 60 s (coded value = −1) to the middle level of 90 s (coded value = 0) enhanced the membrane permeate flux and permeate volume (Figure 4c and Figure 5c), simultaneously [38].

**Figure 6:**
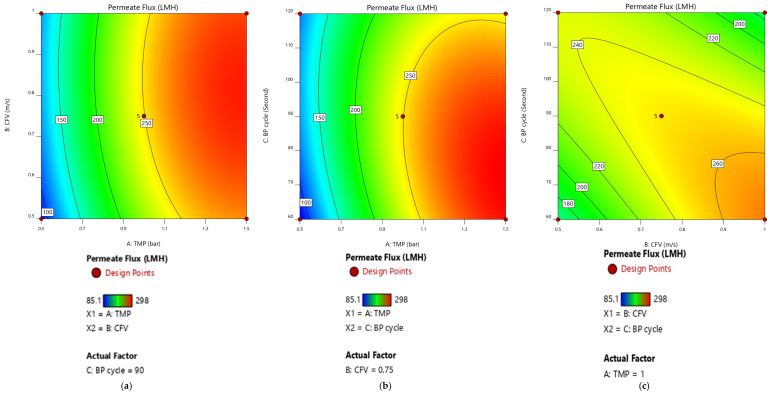
Response surface plot of membrane permeate flux as a function of the independent factors. (**a**) A: TMP and B: CFV at BP cycle= 90 s. (**b**) A: TMP and C: BP cycle at CFV= 0.75 m/s. (**c**) B: CFV and C: BP cycle at TMP = 1 bar.

**Figure 7:**
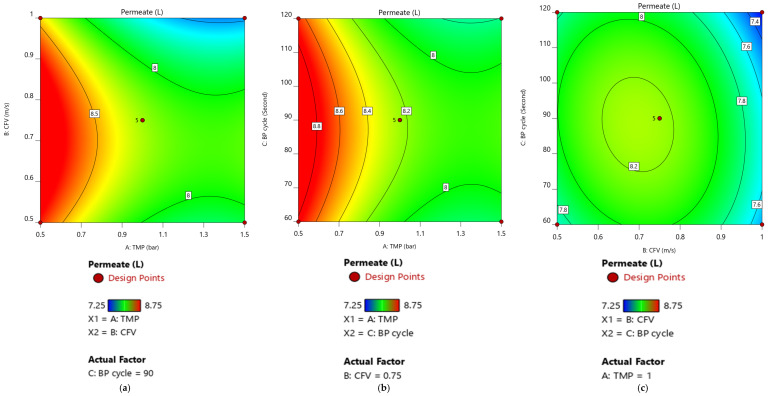
Response surface-plot of membrane permeate volume as a function of the independent factors. (**a**) A: TMP and B: CFV at BP cycle= 90 s. (**b**) A: TMP and C: BP cycle at CFV= 0.75 m/s. (**c**) B: CFV and C: BP cycle at TMP = 1 bar.

The elliptical contour plots represent the interaction of two factors and their impact on the response value (Figure 6 and Figure 7) while maintaining the third variable constant at its central point value. Figure 6a shows that at the central point of the pulsatile cycle at 90 s, the interaction of TMP and CFV is not significant at 95% CI. However, the increase in the range variation of the CFV and TMP or fluctuation of the design point from the central point value of the pulsatile cycle may improve the membrane permeate flux. Figure 6b illustrates the significant interaction of the TMP and Pulsatile cycle at the central point value of the CFV at 0.75 m/s. The plot shows that when TMP is at its upper range, between 1 and 1.5 bar, and the pulsatile cycle is below its central point value, the membrane permeate-flux response value was at its optimum. In comparison, the response value is low when the TMP design configuration is between 0.5 and 1 bar. In Figure 6c, the results indicate that when TMP is maintained at its central point value of 1 bar, the impact of the CFV and the pulsatile cycle is optimum at their central point values of 0.75 m/s and 90 s, respectively. A standard error calculation of the regression was performed to quantify the accuracy of the estimates. Figures S8 and S9 represent the standard error for Figure 6 and Figure 7, respectively. The plot of the standard error of membrane permeate flux (Figure S8) and permeate volume (Figure S9) as a function of the independent factors (TMP, CFV, and pulsatile cycle) is presented. The smaller the standard error, the more accurate is the prediction of the regression models. In Figures S8 and S9, the darker the shading, the greater is the standard error for a given coordinate of an independent-factor response surface.

Figure 7a,b demonstrated a lack of positive interaction, at a 95% confidence interval, between the TMP and CFV at a Pulsatile cycle of 90 s and between the TMP and Pulsatile cycle at a CFV of 0.75 m/s, respectively. This was due to the dominant impact of TMP on the filtration process. At low pressure, the permeate volume reached a higher value of 8.5 L at the pulsatile cycle of 90 s and 8.8 L at a CFV of 0.75 m/s for all values of the CFV and Pulsatile cycle, respectively. However, when the TMP was set at a higher range beyond the central point value of 1 bar, the permeate volume declined for the CFV and Pulsatile variation, respectively. This could be explained by fouling development at the membrane surface due to the dominant impact of the permeation drag force resulting from the applied pressure-driven difference [39,40].

Membrane permeate volume was studied within a range of CFV from 0.5 to 1 m/s, a Pulsatile cycle from 60 to 120 s, and at 1 bar TMP. The results show that the maximum response was achieved at the middle point values of 0.75 m/s and 90 s for the CFV and Pulsatile cycle, respectively (Figure 7c).

**Figure 8:**
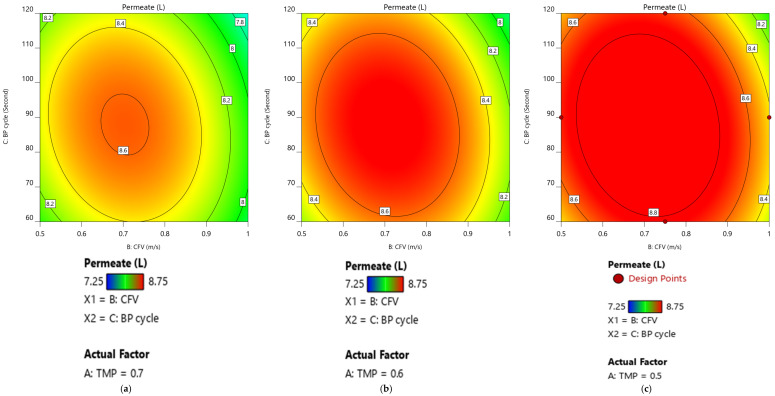
Membrane-permeate volume contour as a function of CFV and Pulsatile cycle at a sequence of TMP values 0.7, 0.6, and 0.5 bar, respectively. (**a**) B: CFV and C: BP cycle at TMP = 0.7 bar. (**b**) B: CFV and C: BP cycle at TMP = 0.6 bar. (**c**) B: CFV and C: BP cycle at TMP = 0.5 bar.

As a result, at 95% CI, the optimum membrane-permeate volume is attained when the cleaning mechanism (CFV, Pulsatile cycle) was in the middle operating value and TMP is below its central point value. Figure 8a–c illustrates the case study for TMP at 0.7, 0.6, and 0.5 bar, respectively.

### 4.6. Optimization and Model Post Analysis

**Figure 9:**
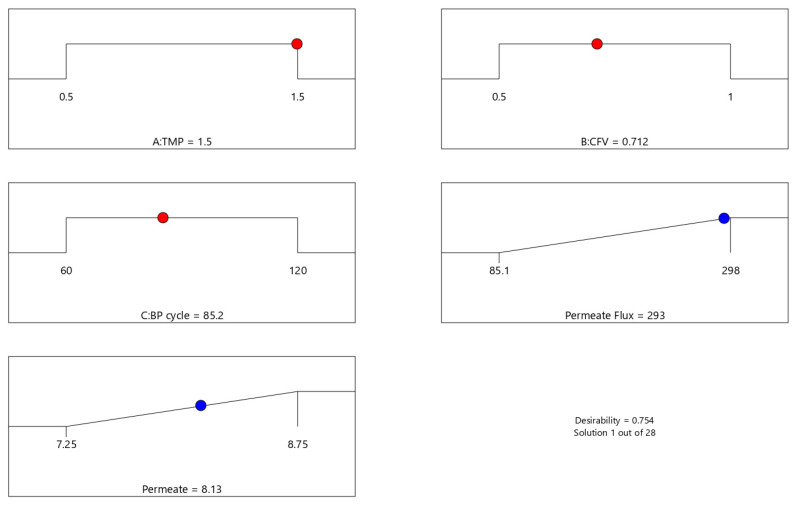
Independent factors ranges, response criteria, and desirability solution for the membrane permeate flux and permeate volume.

**Figure 10:**
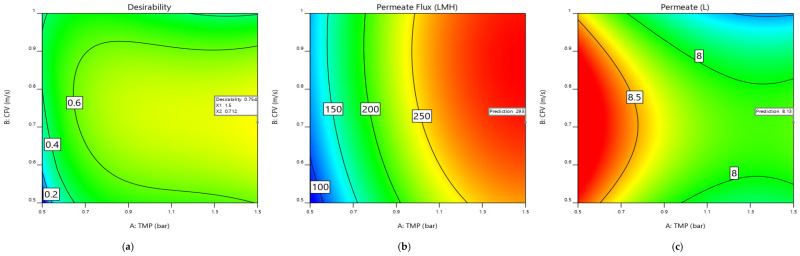
Optimizing the process parameters to maximize the membrane permeate flux and permeate volume. (**a**) Desirability solution for the interaction factors A: TMP, B: CFV, and C: BP cycle at 85.1951 s ≈ 85 s. (**b**) Membrane permeate-flux optimization at the desirability values of A: TMP = 1.5 bar, B: CFV ≈ 0.71 m/s, and C: BP cycle ≈ 85 s. (**c**) Membrane permeate-volume optimization at the desirability values of A: TMP =1.5 bar, B: CFV ≈ 0.71 m/s, and C: BP cycle ≈ 85 s.

As reported earlier (Figure 4 and Figure 5), each independent factor had a specific impact on the response parameters. In contrast, the factors’ interactions may enhance one response but limit the other. The optimal response values were achieved by combining the process parameters levels (TMP, CFV, and Pulsatile cycle) that simultaneously satisfy the maximization criteria for each response. As a result, the desirability function was used as a multi-criteria methodology to optimize this process, identify the optimum parameters, and maximize the response values in Figure 9 and Figure 10, as reported in Tables S2 and S3. All the independent factors and process responses are given a significance level of three and optimized in their range for the maximum membrane-permeate flux and permeate volume, respectively. The results of the numerical optimization are presented in Table 14. The optimized process filtration, called the best run, for predicted permeate flux and membrane-permeate volume responses were 293 LMH and 8.13 L, respectively. The maximum overall desirability obtained was 0.75 for the optimum independent actual values of 1.5 bar, 0.71 m/s, and 85 s, respectively, for the approximate coded factors’ levels of (1, 0, 0). An experimental validation test of the optimal values was performed to examine the results of the predicted response (Table 14). The results showed that when the values of each factor were set at the optimal levels, the predicted and measured permeate flux and permeate volumes were exactly 293 LMH, 8.13 L, and for a desirability factor of 3, the values were 302 LMH, 8.11 L, respectively. A post-analysis has been performed running a normal filtration with no pulsatile process at 1.5 bar and 0.71 m/s for a fair comparison. The obtained permeate flux and volume results were 32 LMH and 7.13 L, respectively. Compared to the same configuration of operating pressure and crossflow velocity of 1.5 bar and 0.71 m/s with a pulsatile cycle of 85 s, the pulsatile process improved the permeability and permeate volume by 89% and 12%, respectively [14,41,42,43].

## 5. Conclusions

This work demonstrated that pulsatile physical cleaning in crossflow filtration systems enhanced membrane filtration and fouling mitigation for a UF ceramic membrane with MWCO of 150 kg/mol. The average feed-oil content of 198 ppm was treated to produce an effluent of less than 3.5 ppm. Membrane fouling was significant without the physical cleaning. However, the implementation of the pulsatile process controls the fouling development. The cleaning performance varied greatly depending on the pulsatile process settings.

The optimization of the pulsatile process in the crossflow filtration system was successfully obtained using the Box–Behnken design method. The established regression models were verified using the analysis of variance (ANOVA) to improve the understanding of the pulsatile mechanism and to investigate the significant impact of the pulsatile cleaning technique on the membrane filtration performance and fouling control. The TMP displays the most substantial effect where a significant interaction occurred between the CFV and pulsatile time for an optimum permeability and permeate volume. In contrast, the interaction effect between the TMP and the pulsatile cycle improved the permeate flux with negligible effect on the net permeate volume due to the filtrate loss caused by the process of flow reversal in the cleaning. The optimized filtration process was achieved at a TMP of 1.5 bar and around the cleaning parameters’ central points, namely a CFV of 0.71 m/s and a pulsatile time of 85 s, respectively.

Moreover, the predicted values for optimal efficiency were experimentally tested and validated for the ceramic membrane, obtaining a steady-state permeability with a flux of 302 LMH and a permeate volume of 8.11 L. In conclusion, implementing the pulsatile process improved the membrane performance and fouling alleviation in a produced water crossflow-filtration system.

## Figures and Tables

**Table 1:** Chemicals and salts used for ceramic cleaning and oil/solvent extraction.

Chemicals	Suppliers	Usage
Sodium hydroxide (NaOH, >95 wt.%)	EMD chemicals	Ceramic cleaning
Phosphoric acid (85%)	BDH Chemicals	Ceramic cleaning
Hydrochloric acid (HCl, SA431-500, 2 N)	Fisher Chemicals	Oil/solvent extraction
Horiba S-316 #100690 Extraction Solvent	Horiba	Oil/solvent extraction

**Table 2:** Average feed characteristics.

Oil Parameters	Feed
Oil Content, ppm	200 ± 3
Chemical Oxygen Demand (COD), mg/L	1274 ± 30
Turbidity, NTU	537 ± 2%
pH	6.117 ± 0.001
Zeta potential, mV	−31 ± 3.0
Mean droplet size, μm	5.2 ± 0.1
Density, g/cc	0.8872 ± 5 × 10^−5^
Viscosity, cP	5.23 ± 1%

**Table 3:** Experimental instruments.

Equipment	Measured Parameter/Function
Horiba Oil Content Analyzer (OCMA-350), Horiba, Kyoto, Japan	Oil content, ppm
Horiba F-55 benchtop meter (Horiba 2003); Horiba, Kyoto, Japan	pH
Hanna turbidity meter (HI 83414, Hanna 2007), Hanna Instruments, Woonsocket, RI, United States	Turbidity, NTU
Hach DR5000 UV-Vis spectrophotometer, Hatch, London, ON, Canada	Chemical Oxygen Demand (COD), mg/L
Zetasizer Nano ZS (ZEN3600, Malvern 2009), Malvern Panalytical Ltd; Malvern, UK	Zeta potential, mV
Zetasizer Nano ZS (ZEN3600, Malvern 2009); Malvern Panalytical Ltd; Malvern, UK	Droplet size, μm
Brookfield viscometer DV-II+Pro, Brookfield Engineering, Middleboro, MA, USA	Viscosity, cP
Anton Paar 5000 DSA 5000 digital densitometer, Anton Paar, Graz, Austria	Density, g/cc
RX-5000 refractometer (ATAGO), ATAGO CO., LTD., Tokyo, Japan	Refractive index (RI)
Waring Commercial MX1000 Series, Waring Commercial, Stamford, CT, USA	Blender

**Table 4:** Ceramic membrane characteristics.

Membrane	Characteristics
Materials	Active layer: Multi-channels titania (TiO_2_)
	Support: Zirconia (ZrO_2_)
Dimensions, mm	25 ± 1 × 305 ± 1
Number of channels	7
Hydraulic diameter of channels, mm	6 ± 0.1
Filtration area, m^2^	0.04186 ± 0.006
Cross-sectional area, m^2^	0.001172 ± 0.006
Pore size/MWCO	150 kg/mol
Porosity	38%
Maximum working pressure	10 bar
Best operating pressure	3 bar
Bursting pressure	>90 bar
pH range	0–14
Max operating temperature	<250 °C
Thermal shock resistance	ΔT instantaneous <60 °C (Temperature difference between liquid and membrane)
Steam sterilization	121 °C—30 min

**Table 5:** Independent factors and their levels.

Variables	Codes	Levels		
		−1	0	+1
TMP (bar)	A	0.50	1	1.50
CFV (m/s)	B	0.50	0.75	1
Pulsatile cycle (s)	C	60	90	120

**Table 6:** Responses parameters.

Response	Name	Units	Analysis	Min	Max	Mean	Std. Dev.	Ratio	Model
R1	Permeate Flux	LMH	Polynomial	85.1	298	210.80	72.13	3.50	Quadratic
R2	Permeate volume	L	Polynomial	7.25	8.75	8.06	0.4497	1.21	Quadratic

**Table 7:** Three Box–Behnken design factors and their corresponding responses.

		Uncoded Factors			Coded Factors			Responses			
Std Run	Run Test Order	TMP	CFV	Pulsatile Cycle	A	B	C	Permeate Flux (J_ni_)		Permeate Volume (Y_ni_)	
								Experimental	Predicted	Experimental	Predicted
		(bar)	(m/s)	(s)				(LMH)	(LMH)	(L)	(L)
8	1	1.5	0.75	120	1	0	1	236	232	7.85	7.85
5	2	0.5	0.75	60	−1	0	−1	89	85	8.73	8.73
6	3	1.5	0.75	60	1	0	−1	298	298	7.91	7.91
7	4	0.5	0.75	120	−1	0	1	108	108	8.65	8.66
10	5	1	1	60	0	1	−1	267	263	7.52	7.52
9	6	1	0.5	60	0	−1	−1	159	159	7.71	7.72
3	7	0.5	1	90	−1	1	0	116	116	8.45	8.45
2	8	1.5	0.50	90	1	−1	0	271	271	7.88	7.87
15	9	1	0.75	90	0	0	0	259	244	8.25	8.24
12	10	1	1	120	0	1	1	178	174	7.25	7.26
4	11	1.5	1	90	1	1	0	286	286	7.43	7.43
17	12	1	0.75	90	0	0	0	252	244	8.26	8.24
1	13	0.5	0.5	90	−1	−1	0	85	85	8.75	8.75
13	14	1	0.75	90	0	0	0	244	248	8.24	8.24
14	15	1	0.75	90	0	0	0	248	244	8.19	8.24
11	16	1	0.5	120	0	−1	1	244	244	7.75	7.75
16	17	1	0.75	90	0	0	0	244	244	8.21	8.24
Best Run		1.5	0.71	85	1	≈0	≈0	302	293	8.11	8.13
Normal filtration		1.5	1	-	-	-	-	35	-	6.85	-

**Table 8:** Feed and permeate characteristics.

		Synthetic Feed		Permeate		Turbidity	
Std	Run	Mean Oil Droplet Size	Oil Content	Oil Content	Rejection	Feed	Permeate
Run	Test Order	(μm)	(ppm)	(ppm)	(%)	(NTU)	(NTU)
8	1	6.5	198	8	96	563	3.36
5	2	5.4	200	5	98	568	0.75
6	3	5.3	195	5	97	565	0.95
7	4	5.8	200	8	96	571	3.31
10	5	6.4	199	4	98	569	0.81
9	6	6.3	196	11	94	558	3.62
3	7	6.9	197	4	98	562	0.67
2	8	4.9	194	5	97	560	2.11
15	9	6.8	193	6	97	562	2.03
12	10	5.5	200	12	94	569	3.78
4	11	5.4	198	6	97	565	2.21
17	12	5.3	200	10	95	570	3.44
1	13	5.5	198	4	98	561	0.93
13	14	4.9	196	8	96	555	3.33
14	15	4.8	198	9	95	563	3.42
11	16	5.3	197	10	95	557	3.46
16	17	5.5	200	11	95	572	3.58
Best Run		5.3	196	3	99	558	0.32
Normal filtration		5.5	198	16	92	562	4.58

**Table 9:** Permeate flux-regression polynomial coefficients in terms of coded and actual factors.

Flux	Coded Factors	Flux	Actual Factors
249.23		−990.24	
86.59	A	672.80	TMP
11.13	B	964.79	CFV
−5.80	C	9.93	BP cycle
−3.87	AB	−30.96	TMP × CFV
−20.32	AC	−1.35	TMP × BP cycle
−43.54	BC	−5.81	CFV × BP cycle
−44.31	A²	−177.58	TMP²
−15.29	B²	−244.58	CFV²
−22.06	C²	−0.03	BP cycle²

**Table 10:** Permeate volume-regression polynomial coefficients in terms of coded factors and actual factors.

Permeate	Coded Factors	Permeate	Actual Factors
8.230		4.091	
−0.439	A	−3.183	TMP
−0.180	B	10.470	CFV
−0.046	C	0.057	BP cycle
−0.038	AB	−0.300	TMP × CFV
0.005	AC	0.0003	TMP × BP cycle
−0.078	BC	−0.010	CFV × BP cycle
0.313	A²	1.250	TMP²
−0.415	B²	−6.640	CFV²
−0.258	C²	−0.0003	BP cycle²

**Table 11:** Statistics for the fitting models.

Membrane Permeate Flux				Membrane Permeate Volume			
Std. Dev.	7.59	R²	0.995	Std. Dev.	0.0447	R²	0.996
Mean	210.80	Adjusted R²	0.989	Mean	8.06	Adjusted R²	0.990
C.V. %	3.60	Predicted R²	0.952	C.V. %	0.5543	Predicted R²	0.946
		Adeq Precision *	36.714			Adeq Precision	44.391

* Adeq precision is used to measure the signal-to-noise ratio. A value higher than four means adequate model discrimination.

**Table 12:** ANOVA for the fit of membrane permeate flux.

Source	Sum of Squares	DF	Mean Square	F-Value	*p*-Value	
**Model**	82,841	9	9205	159.63	<0.0001	significant
A-TMP	59,984	1	59,984	1040	<0.0001	
B-CFV	990	1	990	17.18	0.0043	
C-BP cycle	269	1	269	4.68	0.0674 *	
AB	59.91	1	59.91	1.04	0.3420 *	
AC	1651	1	1651	28.64	0.0011	
BC	7582	1	7582	131.49	<0.0001	
A²	8267	1	8267	143.38	<0.0001	
B²	984	1	984	17.06	0.0044	
C²	2049	1	2049	35.53	0.0006	
**Residual**	404	7	57.66			
Lack of Fit	236	3	78.63	1.88	0.2747	not significant

* not significant at a 95% confidence level.

**Table 13:** ANOVA for the fit of membrane permeate volume.

Source	Sum of Squares	DF	Mean Square	F-Value	*p*-Value	
**Model**	3.22	9	0.3579	179.28	<0.0001	significant
A-TMP	1.54	1	1.54	771.38	<0.0001	
B-CFV	0.2592	1	0.2592	129.83	<0.0001	
C-BP cycle	0.0171	1	0.0171	8.57	0.0221	
AB	0.0056	1	0.0056	2.82	0.1371 *	
AC	0.0001	1	0.0001	0.0501	0.8293 *	
BC	0.0240	1	0.0240	12.03	0.0104	
A²	0.4112	1	0.4112	205.96	<0.0001	
B²	0.7252	1	0.7252	363.23	<0.0001	
C²	0.2792	1	0.2792	139.84	<0.0001	
**Residual**	0.0140	7	0.0020			
Lack of Fit	0.0106	3	0.0035	4.15	0.1015	not significant

* not significant at a 95% confidence level.

**Table 14:** Optimization results for the membrane permeate flux and permeate volume.

Factors Optimized Coded Level	A: TMP (bar)	1.5
	B: CFV (m/s)	0.71
	C: Pulsatile cycle (sec)	85
Predicted responses	Permeate flux (LMH)	293
	Permeate volume (L)	8.13
Overall desirability		0.75

## Data Availability

All data are presented in the article.

## Supplementary Materials

The following supporting information can be downloaded at: https://www.mdpi.com/article/10.3390/membranes12121198/s1. Table S1. Maximum flux values at the beginning of the filtration process for RO water and feed; Table S2. Constraints; Table S3. Desirability Solutions; Figure S1. LabBrain membrane filtration system; Figure S2. From left to right, Bakken oil and produced water feed with 200 ppm oil; Figure S3. Synthetized produced water (oil, water, and SDS surfactant); Figure S4. Contact angles of a Bakken oil droplet at the ceramic membrane surface; Figure S5. Contact angles of a water droplet at the ceramic membrane surface; Figure S6. LabBrain filtration unit P&I diagram system; Figure S7. Membrane permeate flux as a function of time for normal filtration (No pulsatile) (a) and 40 Pulsatile cycle at 60 s (b–e), 90 s (f–j), and 120 s (k–n), respectively; Figure S8. Response surface plot of membrane permeate-flux standard error as a function of TMP, CFV, and Pulsatile cycle; Figure S9. Response surface plot of membrane permeate volume standard error as a function of the TMP, CFV, and Pulsatile cycle.
