# Supplementary material for: High-Frequency Pulsatile Parameterization Study for the Titania Ceramic Membrane Fouling Mitigation in Oily Wastewater Systems Using the Box–Behnken Response Surface Methodology
Source: Membranes (Basel). 2022 Nov 28;12(12):1198. doi: 10.3390/membranes12121198 (PMC9788362; doi:10.3390/membranes12121198)
Supplement: Supplementary file 1 [file membranes-12-01198-s001.zip › membranes-2025028-supplementary.pdf]

membranes

MDPI

Supplementary Material

High-Frequency Pulsatile Parameterization Study for the Titania Ceramic Membrane Fouling Mitigation in Oily Wastewater Systems Using The Box–Behnken Response Surface Methodology

Mohamed Echakouri, Amr Henni * and Amgad Salama

Process Systems Engineering, Produced Water Treatment Laboratory, Faculty of Engineering and Applied Science, University of Regina, Regina, SK S4S 0A2, Canada
* Correspondence: amr.henni@uregina.ca

Table S1. Maximum flux values at the beginning of the filtration process for RO water and feed.

	RO water	Feed, 200 ppm
**Maximum flux, L/m². hr**	478	387

Table S2. Constraints.

Name	Goal	Lower Limit	Upper Limit	Lower Weight	Upper Weight	Importance
A: TMP	is in range	0.5	1.5	1	1	3
B: CFV	is in range	0.5	1	1	1	3
C: BP cycle	is in range	60	120	1	1	3
Flux	maximize	85.1	298	1	1	3
Permeate	maximize	7.25	8.75	1	1	3

Table S3. Desirability Solutions.

Number	TMP	CFV	BP cycle	Permeate Flux	Permeate	Desirability	
**1**	**1.500**	**0.712**	**85.200**	**292.619**	**8.125**	**0.754**	**Selected**
2	1.500	0.713	84.999	292.731	8.125	0.754	
3	1.500	0.711	85.462	292.402	8.126	0.754	
4	1.500	0.713	84.793	292.859	8.124	0.754	
5	1.500	0.710	85.731	292.255	8.127	0.754	
6	1.500	0.708	85.655	292.143	8.127	0.754	
7	1.500	0.719	84.870	293.238	8.122	0.754	
8	1.500	0.709	84.607	292.585	8.125	0.754	
9	1.500	0.719	85.405	292.996	8.123	0.754	
10	1.500	0.709	86.258	291.942	8.128	0.754	
11	1.500	0.705	85.459	291.938	8.128	0.754	
12	1.500	0.705	86.127	291.719	8.129	0.754	
13	1.500	0.713	86.322	292.172	8.127	0.754	
14	1.500	0.707	84.486	292.451	8.125	0.754	
15	1.500	0.703	86.005	291.627	8.129	0.754	
16	1.500	0.722	84.423	293.671	8.120	0.754	
17	1.500	0.713	83.770	293.211	8.122	0.754	
18	1.500	0.725	85.298	293.510	8.120	0.754	
19	1.500	0.718	83.056	293.938	8.118	0.753	
20	1.500	0.717	87.109	292.018	8.126	0.753	
21	1.500	0.700	87.338	290.834	8.131	0.753	
22	1.500	0.729	85.230	293.784	8.118	0.753	
23	1.500	0.731	85.851	293.591	8.118	0.753	
24	1.500	0.690	86.992	290.217	8.132	0.753	
25	1.500	0.727	79.769	295.700	8.102	0.750	
26	1.500	0.750	86.146	294.497	8.105	0.749	
27	1.500	0.753	85.176	295.332	8.101	0.748	
28	1.500	0.692	94.374	286.183	8.123	0.741	

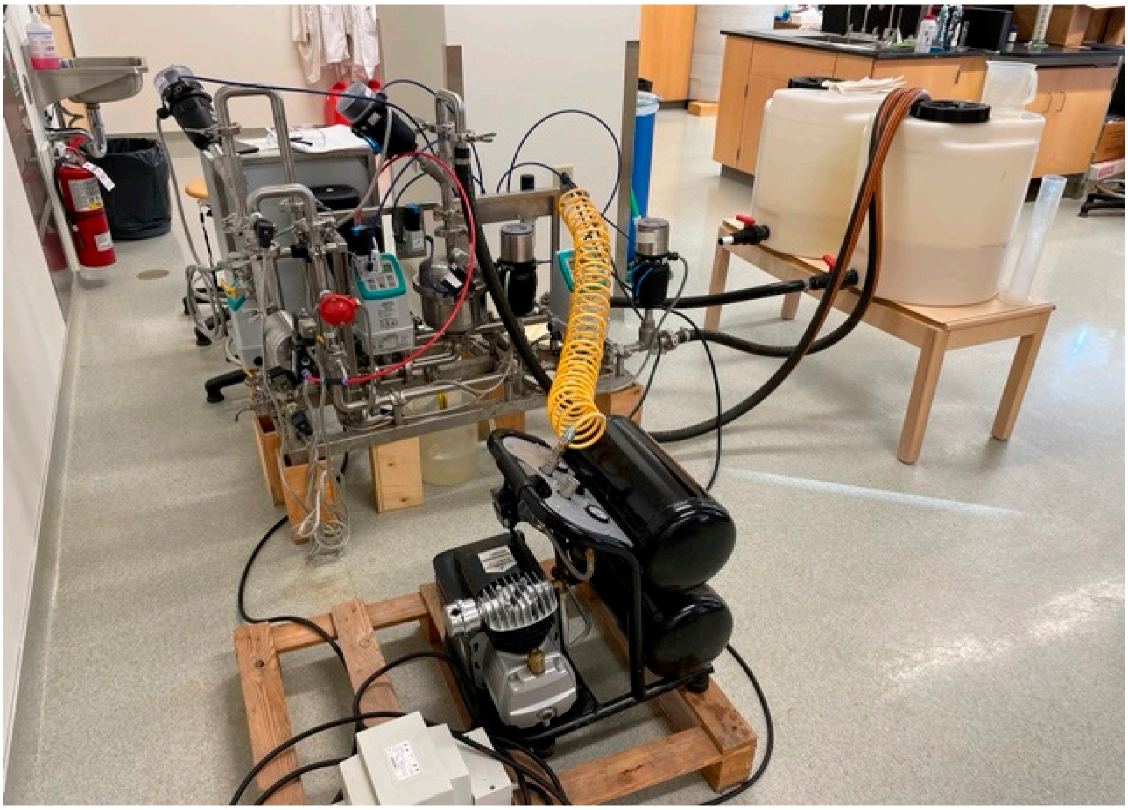

Figure S1. LabBrain membrane filtration system.

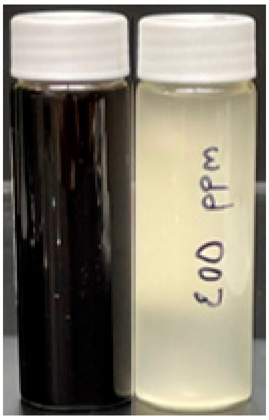

Figure S2. From left to right, Bakken oil and produced water feed with 200 ppm oil.

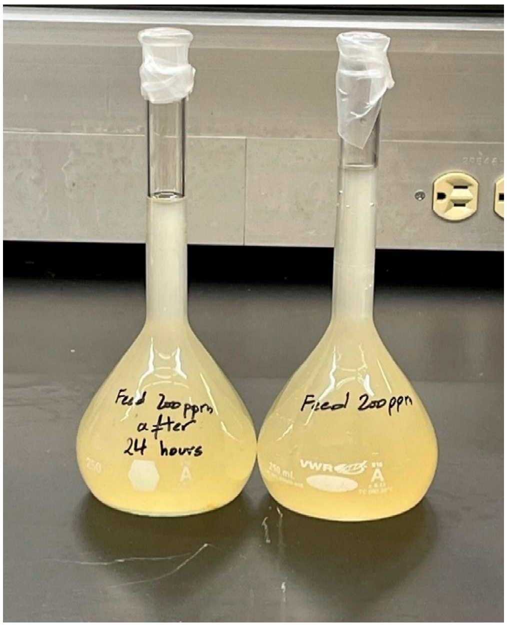

Figure S3. Synthetized produced water (oil, water, and SDS sulfactant).

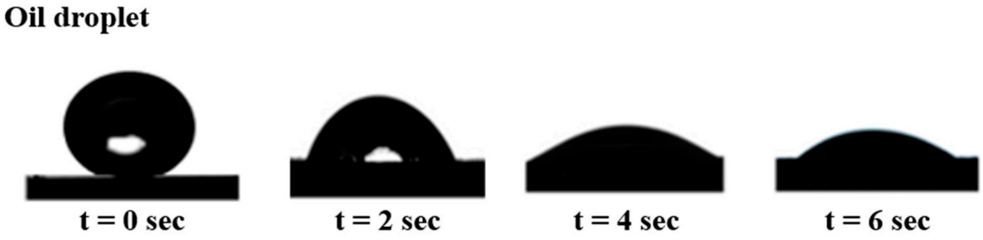

Figure S4. Contact angles of a Bakken oil droplet at the ceramic membrane surface.

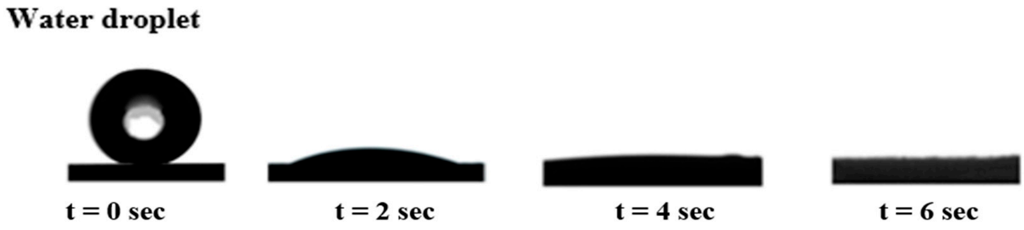

Figure S5. Contact angles of a water droplet at the ceramic membrane surface.

Symbol				Legend			
	**Pump**		**Interface**	**V**	**Valve**	BP	Back-pulse
	Ball valve		Check valve	P	Pump	BF	Backflush
	Flowmeter		Membrane Module	PT	Pressure Transmitter		
	Pressure gauge	B	Back-pulse Hammer	TT	Temperature Transmit-ter		
	Auto on/off	B	Backflush container	FIT	Flowmeter Transmitter and Indicator		
	Auto regulating valve	T	Thermometer	ME	Membrane Housing		

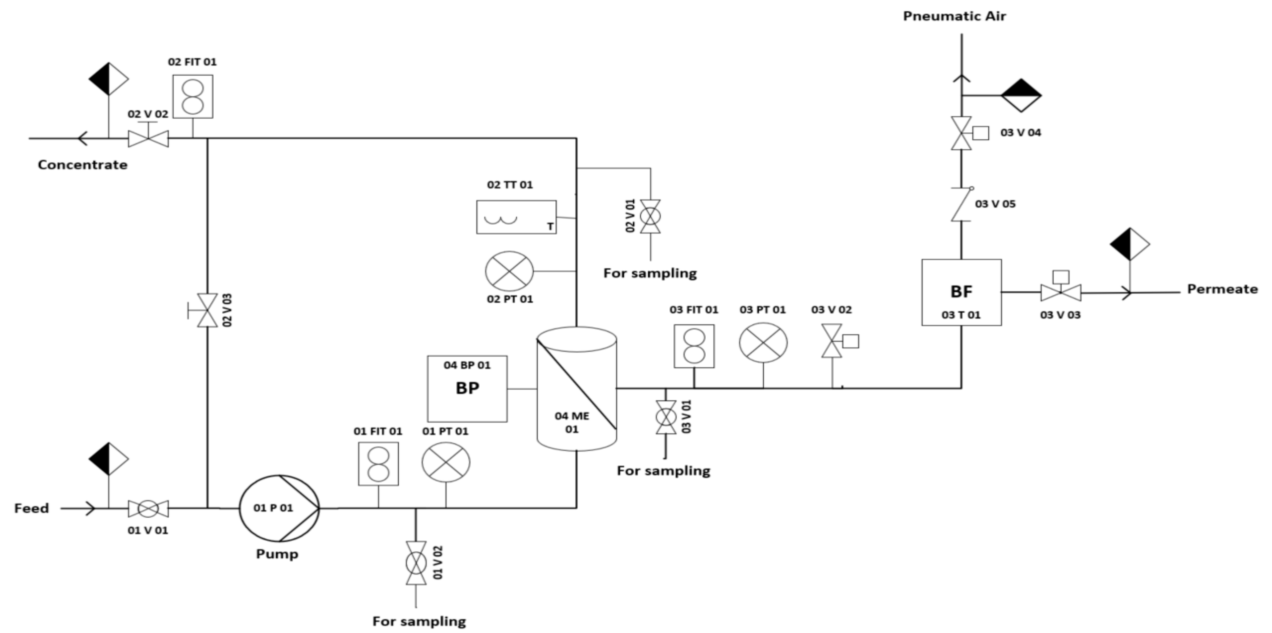

Figure S6. LabBrain filtration unit P&I diagram system.

(a)

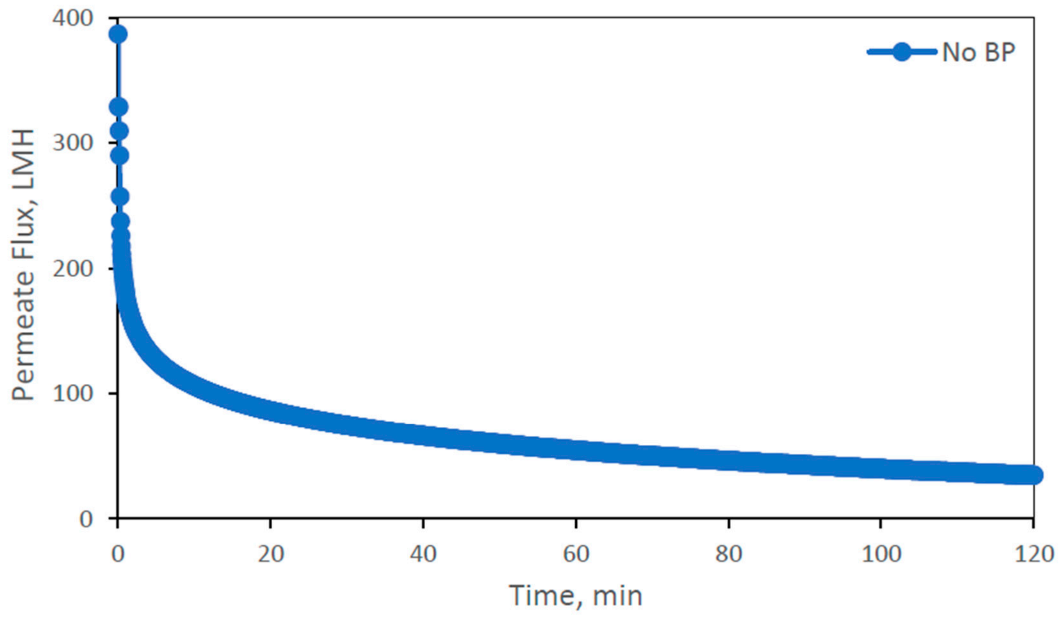

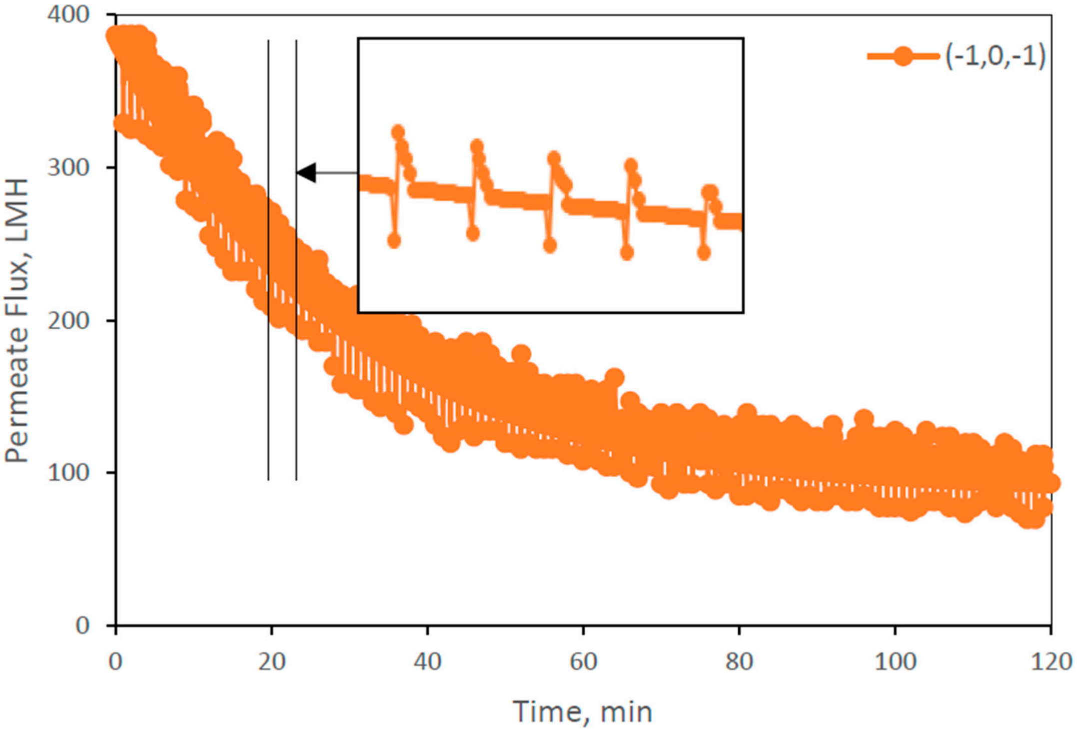
400
300
200
100
0
0
20
40
60
80
100
120
Time, min
Permeate Flux, LMH
(-1,0,-1)

(b)

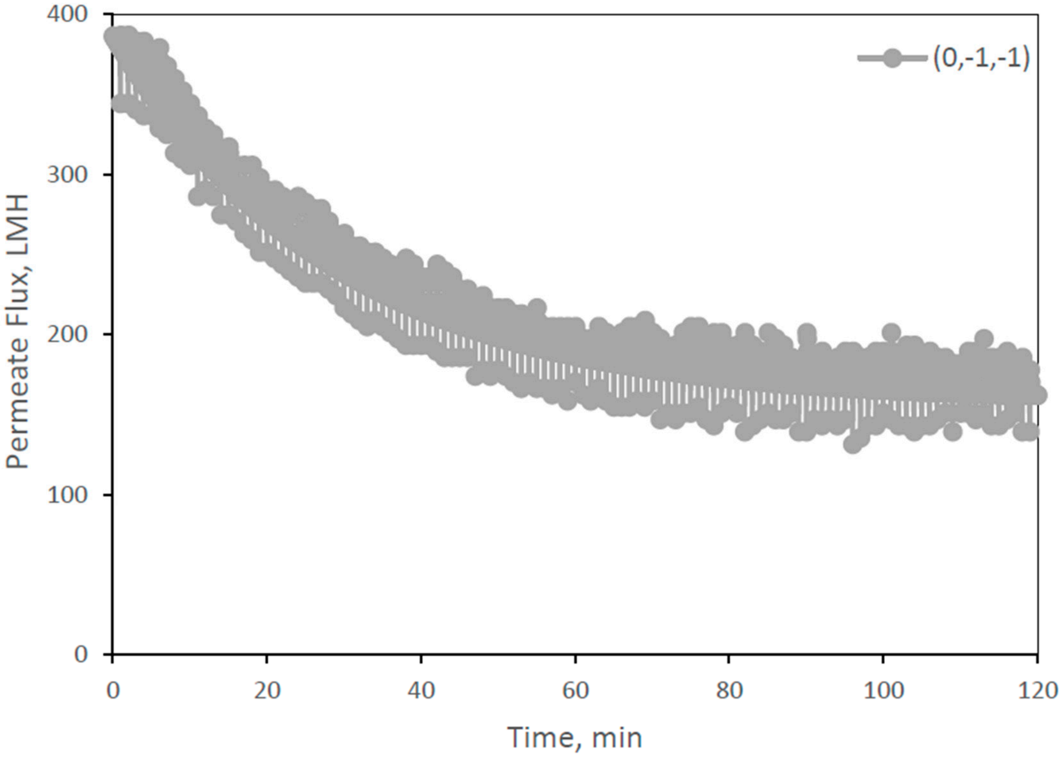
400
300
200
100
0
0
20
40
60
80
100
120
Time, min
Permeate Flux, LMH
(0,-1,-1)

(c)

(d)

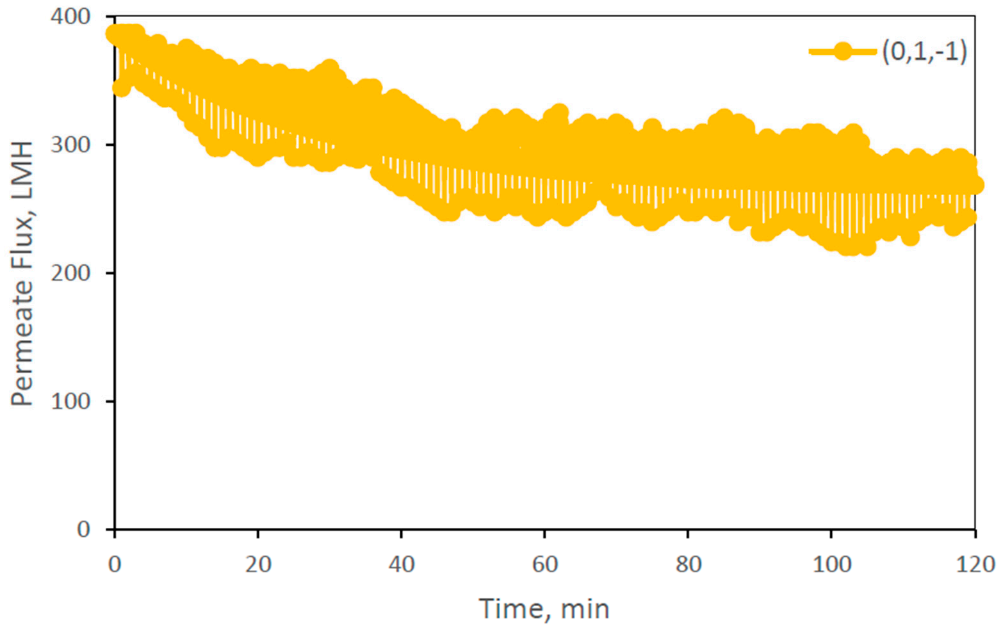

400
300
200
100
0
Permeate Flux, LMH
0
20
40
60
80
100
120
Time, min
(0,1,-1)

(e)

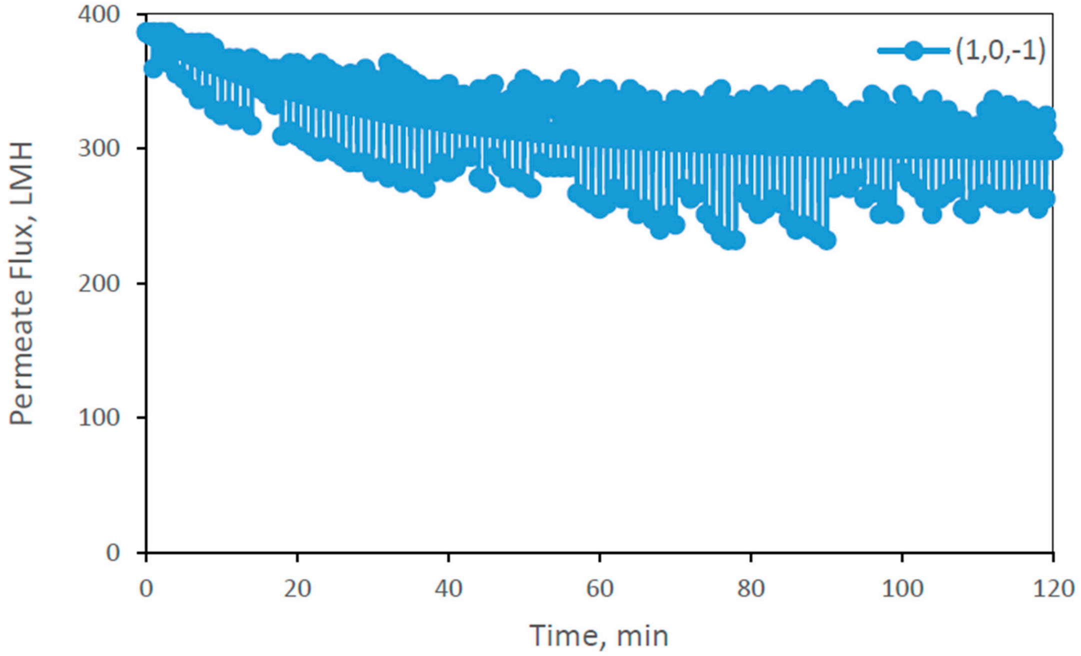

400
300
200
100
0
Permeate Flux, LMH
0
20
40
60
80
100
120
Time, min
(1,0,-1)

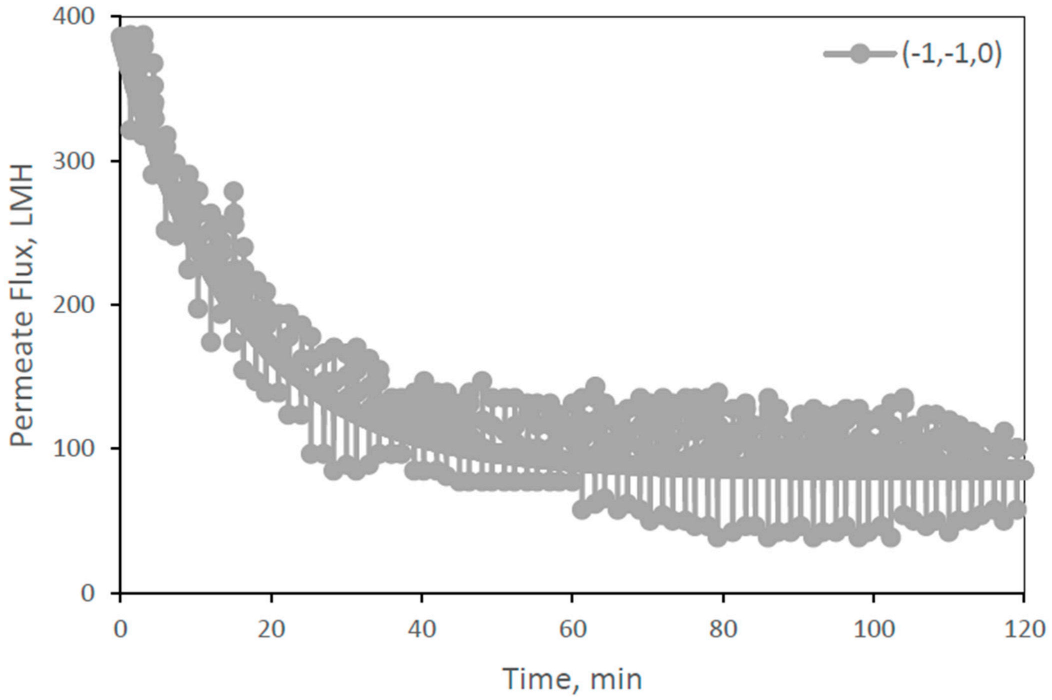
(-1,-1,0)
Permeate Flux, LMH
Time, min
0
100
200
300
400
0
20
40
60
80
100
120

(f)

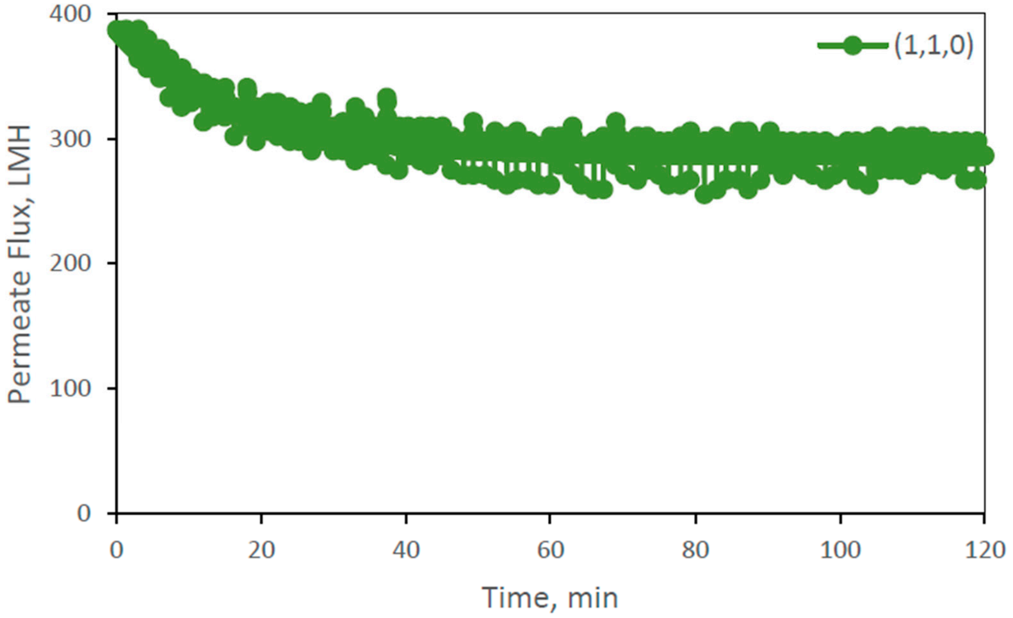
(1,1,0)
Permeate Flux, LMH
Time, min
0
100
200
300
400
0
20
40
60
80
100
120

(g)

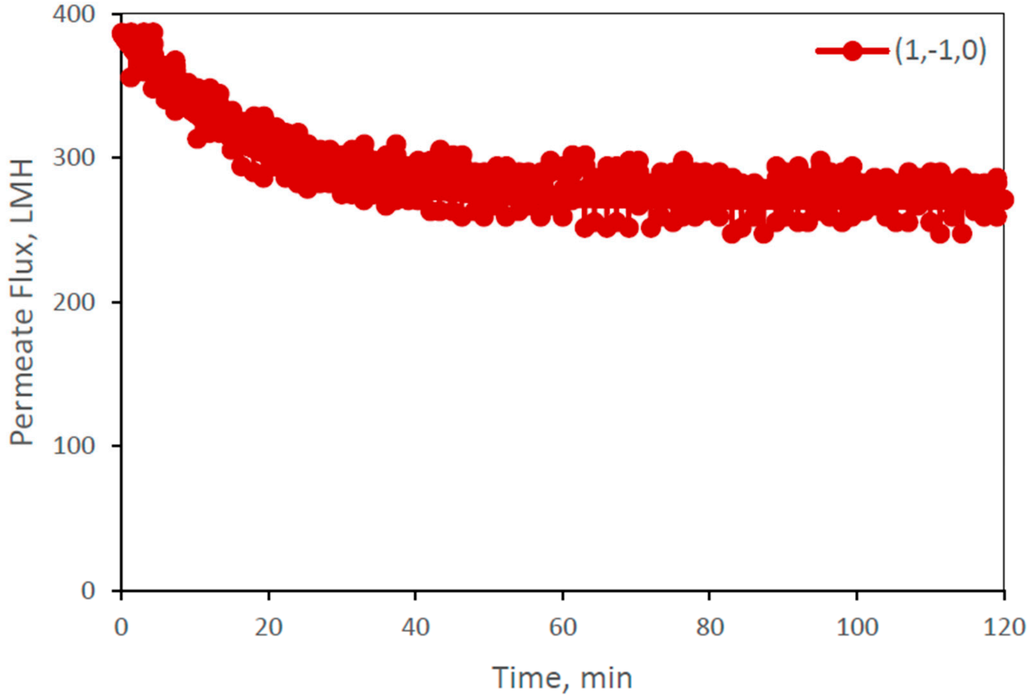

(1,-1,0)
Permeate Flux, LMH
Time, min
0
100
200
300
400
0
20
40
60
80
100
120

(h)

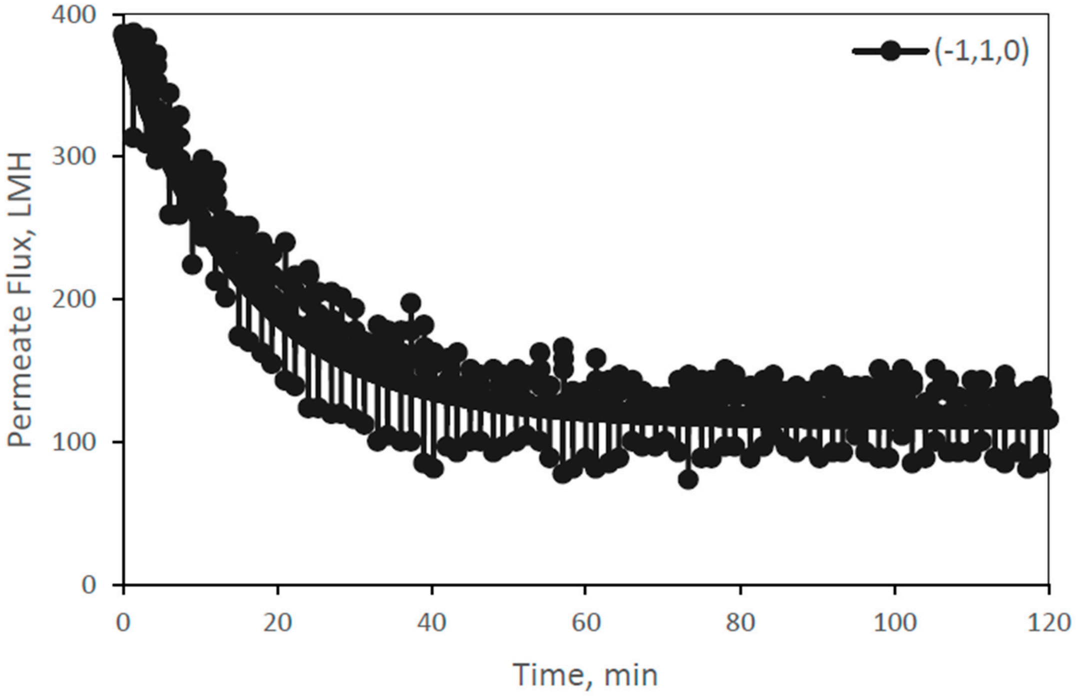

(-1,1,0)
Permeate Flux, LMH
Time, min
0
100
200
300
400
0
20
40
60
80
100
120

(i)

(j)

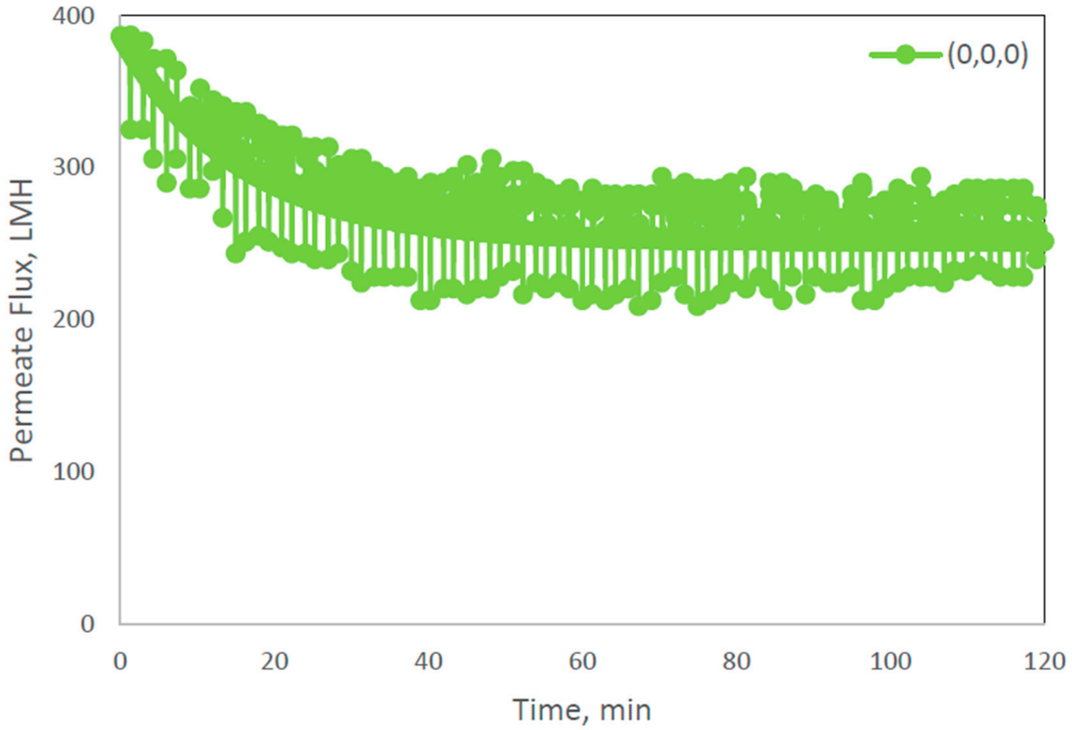
400
300
200
100
0
Permeate Flux, LMH
0
20
40
60
80
100
120
Time, min
(0,0,0)

(k)

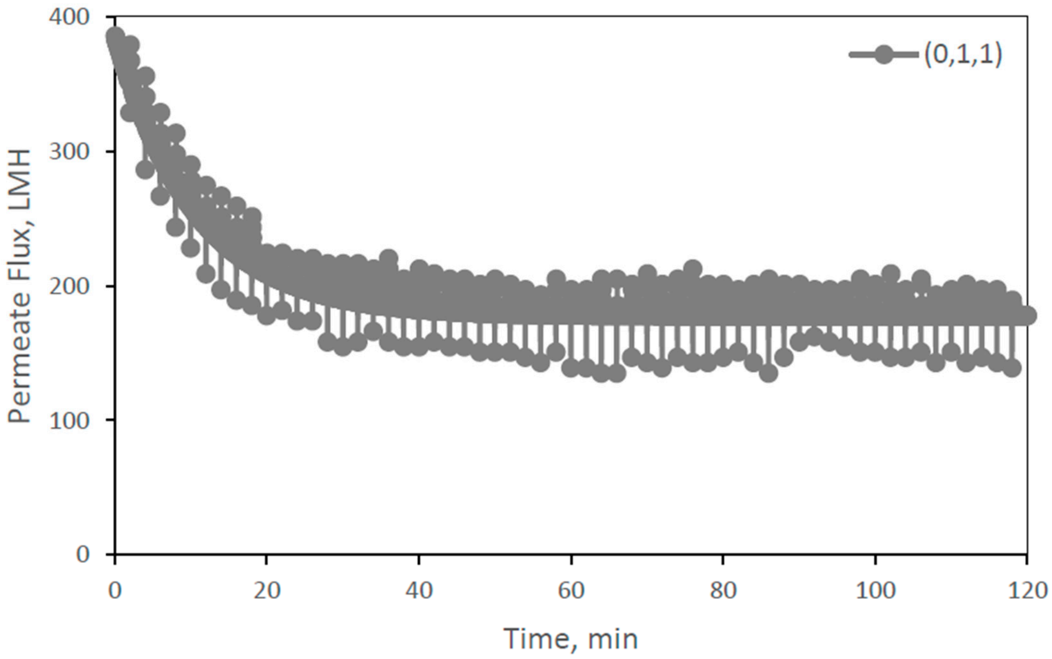
400
300
200
100
0
Permeate Flux, LMH
0
20
40
60
80
100
120
Time, min
(0,1,1)

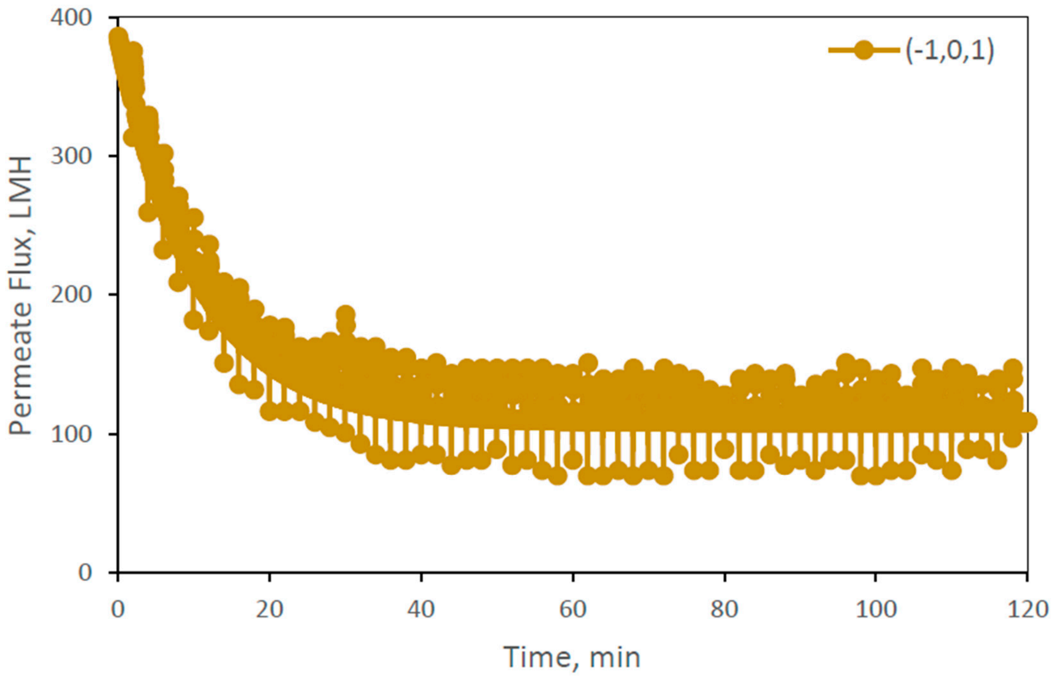
(-1,0,1)
Permeate Flux, LMH
Time, min
0
100
200
300
400
0
20
40
60
80
100
120

(l)

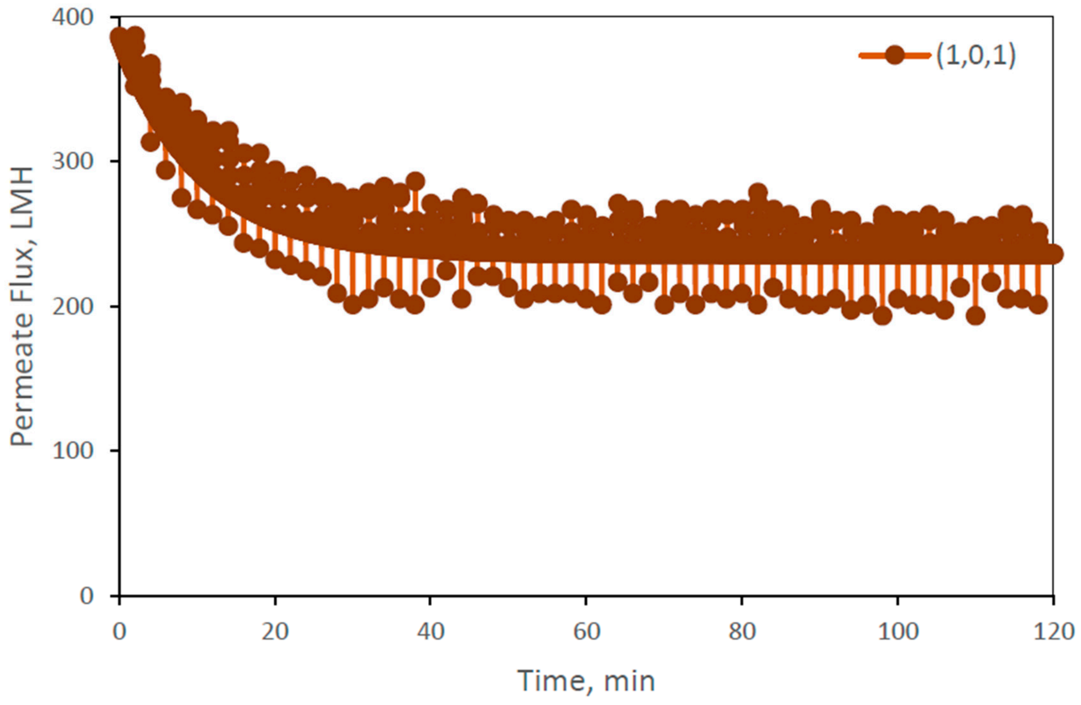
(1,0,1)
Permeate Flux, LMH
Time, min
0
100
200
300
400
0
20
40
60
80
100
120

(m)

(n)

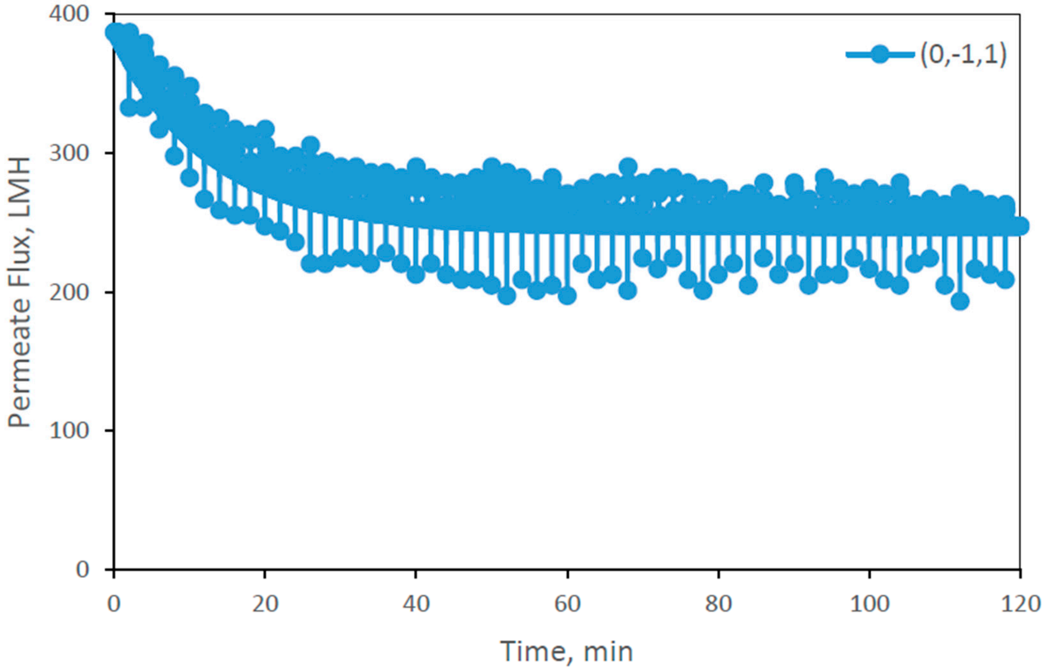

Figure S7. Membrane permeate flux as a function of time for normal filtration (No pulsatile) (a) and Pulsatile cycle at 60 sec (b, c, d, and e), 90 sec (f, g, h, i, and j), and 120 sec (k, l, m, and n), respectively.
